# Interstitial Photothermal Therapy Generates Durable Treatment Responses in Neuroblastoma

**DOI:** 10.1002/adhm.202201084

**Published:** 2022-08-18

**Authors:** Debbie K. Ledezma, Preethi B. Balakrishnan, Anshi Shukla, Jacob A. Medina, Jie Chen, Emily Oakley, Catherine M. Bollard, Gal Shafirstein, Mario Miscuglio, Rohan Fernandes

**Affiliations:** ^1^ The George Washington Cancer Center The George Washington University 800 22nd St NW, 8300 Science and Engineering Hall Washington DC 20052 USA; ^2^ The Institute for Biomedical Sciences The George Washington University 2300 Eye Street NW, Ross Hall Room 561 Washington DC 20037 USA; ^3^ Photodynamic Therapy Center Roswell Park Comprehensive Cancer Center Department of Cell Stress Biology Roswell Park, Elm and Carlton Streets Buffalo NY 14263 USA; ^4^ Center for Cancer and Immunology Research Children's National Hospital 111 Michigan Ave NW Washington DC 20010 USA; ^5^ Department of Electrical and Computer Engineering The George Washington University 800 22nd St NW, 5000 Science and Engineering Hall Washington DC 20052 USA; ^6^ Department of Medicine The George Washington University 2150 Pennsylvania Avenue, NW, Suite 8‐416 Washington DC 20037 USA

**Keywords:** diffuser, interstitial laser, neuroblastoma, photothermal therapy, Prussian blue nanoparticles, thermal dose

## Abstract

Photothermal therapy (PTT) represents a promising modality for tumor control typically using infrared light‐responsive nanoparticles illuminated by a wavelength‐matched external laser. However, due to the constraints of light penetration, PTT is generally restricted to superficially accessible tumors. With the goal of extending the benefits of PTT to all tumor settings, interstitial PTT (I‐PTT) is evaluated by the photothermal activation of intratumorally administered Prussian blue nanoparticles with a laser fiber positioned interstitially within the tumor. This interstitial fiber, which is fitted with a terminal diffuser, distributes light within the tumor microenvironment from the “inside‐out” as compared to from the “outside‐in” traditionally observed during superficially administered PTT (S‐PTT). I‐PTT improves the heating efficiency and heat distribution within a target treatment area compared to S‐PTT. Additionally, I‐PTT generates increased cytotoxicity and thermal damage at equivalent thermal doses, and elicits immunogenic cell death at lower thermal doses in targeted neuroblastoma tumor cells compared to S‐PTT. In vivo, I‐PTT induces significantly higher long‐term tumor regression, lower rates of tumor recurrence, and improved long‐term survival in multiple syngeneic murine models of neuroblastoma. This study highlights the significantly enhanced therapeutic benefit of I‐PTT compared to traditional S‐PTT as a promising treatment modality for solid tumors.

## Graphical abstract

Nanoparticle‐based photothermal therapy (PTT) is an effective method for tumor eradication. However, its conventional application through superficial laser administration (S‐PTT) from the “outside‐in” limits PTT to accessible tumors. Here, interstitial PTT (I‐PTT), which builds on clinical, interstitial laser approaches using Prussian blue nanoparticles to treat tumors from the “inside‐out” yielding significantly improved treatment outcomes over S‐PTT is described.

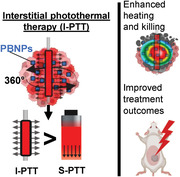

## 1 Introduction

Photothermal therapy (PTT) has emerged as a promising treatment modality for cancer in the search for a precise and effective anticancer therapy. PTT is a thermal therapy in which nanoparticle‐based photothermal agents irradiated with a specific wavelength of light absorb the incident light and convert it to heat, generating hyperthermic or ablative thermal stress in targeted tumor cells or tissue.^[^ ^1^, ^2^, ^3^ ^]^ Several nanoparticles, including Prussian blue nanoparticles (PBNPs) studied by our group, have been utilized as an agent for thermal therapy of tumors, alone and in combination with other therapies such as chemotherapy^[^ ^4^, ^5^, ^6^, ^7^ ^]^ and immunotherapy.^[^ ^8^, ^9^, ^10^, ^11^, ^12^, ^13^, ^14^, ^15^ ^]^ Compared to traditional cancer treatments, nanoparticle‐based PTT is highly localized, minimally invasive, and generates lower systemic side effects.

Despite this promise, the range of applicability of this technique is limited by the delivery mechanism of the light to the nanoparticles. In fact, it typically involves an external laser beam in the IR wavelength range, whose penetration depth through tissue confines PTT to superficially accessible tumors. For the purpose of this study, we define this superficially administered mode of PTT as superficial PTT or S‐PTT. Consequently, tumor tissue beyond the range accessible to S‐PTT has limited‐to‐no light exposure and incomplete heating, potentially leaving untreated, live tumor cells to drive tumor recurrence.^[^ ^16^ ^]^ While increasing the PTT temperature is a consideration to target and eliminate residual tumor cells to minimize recurrence, this comes with a risk of high thermal damage to critical, nontumor tissue. Therefore, to overcome the limitations of S‐PTT, we propose to interstitially administer PTT (I‐PTT) as a clinically relevant approach to treat tumor locations not accessible to external lasers.

I‐PTT is inspired by and proposes to build on the antitumor responses observed during laser interstitial thermal therapy (LITT) and interstitial photodynamic therapy (PDT). LITT is a minimally invasive approach in which optical fibers are inserted into tumors to deliver laser light for thermal ablation under magnetic resonance imaging (MRI) guidance,^[^ ^17^, ^18^, ^19^, ^20^ ^]^ allowing for precise treatment of inaccessible lesions like brain metastases and gliomas.^[^ ^21^, ^22^, ^23^, ^24^ ^]^ In interstitial PDT, the interstitially placed optical fibers deliver laser light to illuminate and activate photosensitizers that induce reactive oxygen species deep within tumors to improve antitumor responses.^[^ ^25^, ^26^ ^]^ While I‐PTT similarly illuminates tumors from the inside‐out as LITT and PDT, PTT uses thermally active nanoparticles that are absent in both LITT and PDT. Importantly, the presence of nanoparticles during PTT facilitates the capture of released antigens and proteins in the tumor microenvironment after treatment, driving improved local and systemic immune‐mediated antitumor responses.^[^ ^27^, ^28^ ^]^

To administer I‐PTT, we utilize PBNPs because of their ability to function as effective PTT agents.^[^ ^11^, ^29^, ^30^ ^]^ Further, Prussian blue capsules are Food and Drug Administration (FDA) approved for elimination of radioactive and nonradioactive cesium and thallium, indicating its safety profile for clinical application.^[^ ^31^, ^32^ ^]^ A previous study has also demonstrated that PBNPs have no significant toxicity when intravenously administered in vitro and in vivo.^[^ ^33^ ^]^ Additionally, PBNPs have been modified to function as imaging agents, including in MRI and photoacoustic imaging applications.^[^ ^34^, ^35^, ^36^ ^]^ Besides its photothermal and imaging capabilities, PBNPs have been used for the delivery of chemotherapy or immunotherapeutic agents.^[^ ^4^, ^37^, ^38^ ^]^ We have previously demonstrated that PBNP‐PTT can elicit immunogenic cell death (ICD),^[^ ^12^, ^13^, ^39^ ^]^ a type of cell death that releases damage‐associated molecular patterns to promote a favorable antitumor immune response. In light of these many advantages and capabilities, we utilize PBNPs for administering I‐PTT instead of alternative nanoparticles.

**Figure 1:**
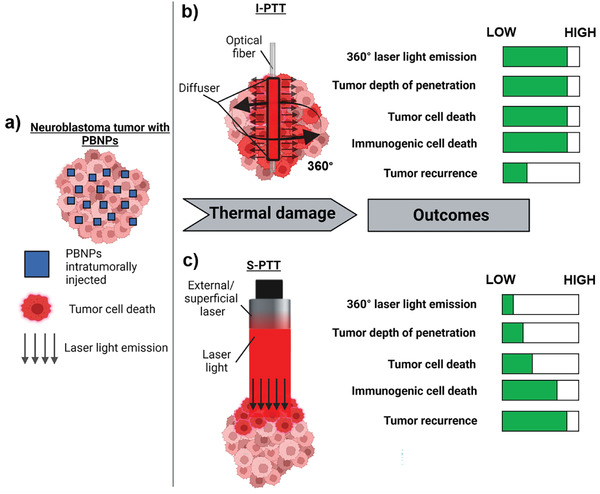
Schematic demonstrating the advantages of interstitial PTT (I‐PTT) for treating neuroblastoma using Prussian blue nanoparticles (PBNPs) compared with external/superficial PTT (S‐PTT). a) PBNPs are i.t. injected into neuroblastoma tumors. b) During I‐PTT, an optical fiber fitted with a terminal diffuser delivers laser light directly into the tumor volume, activating the i.t. PBNPs resulting in more uniform and efficient tumor heating. c) During S‐PTT, the external laser beam illuminates the tumor from the surface, activating the i.t. PBNPs there, thus confining the heat closer to the surface of the tumor. The envisioned treatment effects generated by I‐PTT and S‐PTT are compared in green. Consequently, I‐PTT is expected to result more robust treatment outcomes compared with S‐PTT.

In this study, we compare the photothermal effects of I‐PTT with S‐PTT in models of neuroblastoma. Neuroblastoma is a pediatric cancer that accounts for 15% of childhood cancer‐related deaths. It presents, particularly for high‐risk patients, with tumor recurrence in 50% of cases after intensive, standards‐of‐care treatment regimens.^[^ ^40^, ^41^, ^42^, ^43^, ^44^ ^]^ Since high‐risk neuroblastoma tumors are typically deep‐seated, these patients could potentially benefit from interstitial treatment strategies such as I‐PTT. In our studies, we used Neuro2a (N2A) and TH‐MYCN 9464D (referred to as 9464D hereafter) murine models of neuroblastoma. Specifically, for this comparative study, we applied I‐PTT to subcutaneous neuroblastoma tumors rather than deep‐seated tumors as it allowed us to study the antitumor effects of interstitial laser light delivery without the need to circumvent major organs/tissues. To administer I‐PTT, we coupled an external beam laser to an optical fiber terminating in a cylindrical diffuser to interstitially deliver light to a targeted treatment area (in vitro) or tumor (in vivo). The neuroblastoma tumors were first intratumorally (i.t.) injected with PBNPs (**Figure** **1**a). For I‐PTT, an optical fiber with a terminal diffuser was placed into the tumor (Figure 1b). For S‐PTT, an external/superficial laser was utilized (Figure 1c). We hypothesize that I‐PTT enables a larger volume of illumination and improves depth of penetration in treated tumors resulting in increased tumor cell death, ICD, complete tumor regression, and lower tumor recurrence, when compared to S‐PTT. Through these studies, we seek to answer if I‐PTT significantly improves the beneficial thermal effects and systemic antitumor responses of S‐PTT in addition to providing access to tumors not currently accessible by S‐PTT.

## 2 Results

### 2.1 I‐PTT Improves the Efficiency and Distribution of Heat within a Target Treatment Area

**Figure 2:**
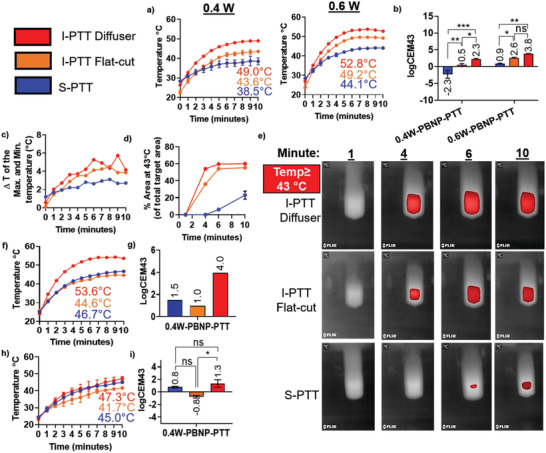
I‐PTT using an optical fiber fitted with a cylindrical terminal diffuser improves the efficiency and distribution of heat within a target treatment area. a) PBS containing 0.1 mg mL^−1^ PBNPs was illuminated at input laser powers of 0.4 and 0.6 W using I‐PTT Diffuser, I‐PTT Flat‐cut, and S‐PTT. Temperatures were measured every minute for 10 min with a thermal camera. b) The thermal doses (logCEM43) achieved using the various PTT configurations calculated from the temperature versus time curves in panel (a) for each laser power. c) The standard deviation of temperatures attained within the sample area during the 10 min treatment with I‐PTT Diffuser, I‐PTT Flat‐cut, or S‐PTT at an input laser power of 0.4 W. d) Ratio of the area at or above 43 °C (red) compared to total target area (light gray + red), indicating a larger area of heating when using I‐PTT (both “Diffuser” and “Flat‐cut”) compared to S‐PTT, which was maintained over the 10 min duration of heating. e) Representative thermal images of the target area containing 0.1 mg mL^–1^ PBNPs illuminated at 0.6 W by I‐PTT Diffuser, I‐PTT Flat‐cut, and S‐PTT, showing the target area (light gray) and the portion of it at or above 43 °C (red), at minutes 1, 4, 6, and 10 of PTT. f) 0.1 mg mL^–1^ PBNPs were added to 2% agarose and the obtained gels were illuminated at an input laser power of 0.4 W with I‐PTT Diffuser, I‐PTT Flat‐cut, or S‐PTT, and temperatures were measured every minute for 10 min with a thermal camera. g) The thermal doses achieved using the various PTT configurations in the 2% agarose gels, calculated from the temperature versus time curve in panel (f). h) 0.1 mg mL^–1^ PBNPs were added to 3.0 mg mL^–1^ Matrigel and illuminated at an input laser power of 0.4 W with I‐PTT Diffuser, I‐PTT Flat‐cut, or S‐PTT, and temperatures were measured every minute for 10 min with a thermal camera. i) The thermal doses achieved using the various PTT configurations in Matrigel, calculated from the temperature versus time curves in panel (h). Values represent means ± st. dev., *p < 0.05, **p < 0.01, ***p <0.001, ****p <0.0001 (ANOVA).

We conducted studies comparing the efficiency and distribution of heating using I‐PTT fitted with a terminal cylindrical diffuser (I‐PTT Diffuser) or a flat‐cut end fiber (I‐PTT Flat‐cut), or using S‐PTT. The photographs showing the setup built in‐house for performing I‐PTT using either a diffuser or a flat‐cut end fiber for in vitro and in vivo studies, and the difference in the light distribution from the two optical fibers are explained in detail in the Experimental and Supporting Information sections (Figures S1 and S2, Supporting Information). The PBNPs used in the study were 50–70 nm in size with a zeta potential of around −30 mV. The nanoparticles maintained consistent sizes and charges (as of Day 771), indicating that PBNPs are stable even after several months and years of synthesis and storage (Figure S3, Supporting Information). Additionally, to ensure that the PBNPs were not aggregating, the polydispersity index (PDI) was consistently measured to be ≈0.2 at several time points after synthesis, indicating that the PBNPs had uniform and consistent size distributions. PBNPs heated using S‐PTT reached final temperatures of 38.5 and 44.1 °C after 10 min at 0.4 and 0.6 W laser powers, respectively. In contrast, I‐PTT Diffuser and I‐PTT Flat‐cut heated to final temperatures of 49.0 and 52.8 °C, and 43.6 and 49.2 °C, respectively (**Figure** **2**a). This advantage in heating with I‐PTT Diffuser is reflected in the higher thermal dose attained, expressed as the logarithm of the cumulative equivalent minutes at 43 °C (logCEM43; more details on thermal dose calculations appear in the Experimental Section 5, In vitro *PBNP‐PTT*).^[^ ^45^ ^]^ I‐PTT Diffuser achieved thermal doses of 2.3 and 3.8 at input laser powers 0.4 and 0.6 W, respectively, which were significantly greater than the −2.3 and 0.9 achieved for S‐PTT and 0.5 and 2.6 achieved for I‐PTT Flat‐cut, respectively (Figure 2b). Analysis of the thermal images indicated that while the heating was localized only to the target area (i.e., PBNP suspension), the temperature difference from the maximum and minimum temperatures in the heating area was higher for I‐PTT Diffuser and I‐PTT Flat‐cut of up to ≈4–5 °C compared to ≈2–2.5 °C for S‐PTT at 0.6 W (Figure 2c). We further analyzed the thermal images to determine the percent of the target treatment area with temperatures of at least 43 °C. A temperature of 43 °C was chosen to represent sections of the target area subjected to temperatures above the thermal breakpoint for hyperthermia (43–49 °C) or ablative heating (≥50 °C).^[^ ^46^ ^]^ After 4 min, treatment with the I‐PTT Diffuser resulted in 54% of the target area at or above 43 °C compared to 36% achieved with I‐PTT Flat‐cut, while S‐PTT heating remained under 43 °C at this time point (Figure 2d). By minute 10, both the I‐PTT Diffuser group and I‐PTT Flat‐cut group had 55–60% of the target area at or above 43 °C, while S‐PTT only had 23% of its target area at or above 43 °C. The thermal images visually demonstrate the target area with at least 43 °C (Figure 2e). Overall, these visual analyses indicate that there is significantly improved heat distribution when using I‐PTT Diffuser or I‐PTT Flat‐cut.

The heating characteristics using these PTT configurations were then compared in aqueous phantoms of agarose and Matrigel to better represent physiological environments, such as the tumor microenvironment and extracellular matrix. I‐PTT Diffuser heated to a higher final temperature of 53.6 °C compared to 44.6 °C for I‐PTT Flat‐cut and 46.7 °C for S‐PTT (Figure 2f), resulting in a higher thermal dose in 2% agarose containing 0.1 mg mL^–1^ PBNP (Figure 2g). Interestingly, the treatments all heated to closer final temperatures of 47.3, 41.7, and 45.0 °C for I‐PTT Diffuser, I‐PTT Flat‐cut, and S‐PTT, respectively, in Matrigel containing 0.1 mg mL^–1^ PBNPs (Figure 2h). Although this led to a slightly higher thermal dose in the I‐PTT Diffuser group (1.3) compared to the S‐PTT group (0.8), this was not statistically significant. I‐PTT Flat‐cut had a lower thermal dose (−0.8; Figure 2i). These photothermal heating studies in both suspension and gel phantoms suggest that I‐PTT Diffuser improves heating by increasing the thermal dose by 1.2–3.0 (logCEM43) over I‐PTT Flat‐cut and by 0.5–4.6 (logCEM43) over S‐PTT, while demonstrating improved localized heating distribution by 32% compared to S‐PTT. Compared to I‐PTT Flat‐cut, I‐PTT Diffuser consistently generated more efficient heating and heat distribution at the same input laser power, and thus for the following studies, we only compared I‐PTT Diffuser (henceforth referred to as I‐PTT) with S‐PTT without additional reference to I‐PTT Flat‐cut.

### 2.2 I‐PTT Triggers Increased Cytotoxicity and Thermal Damage in Targeted Tumor Cells

**Figure 3:**
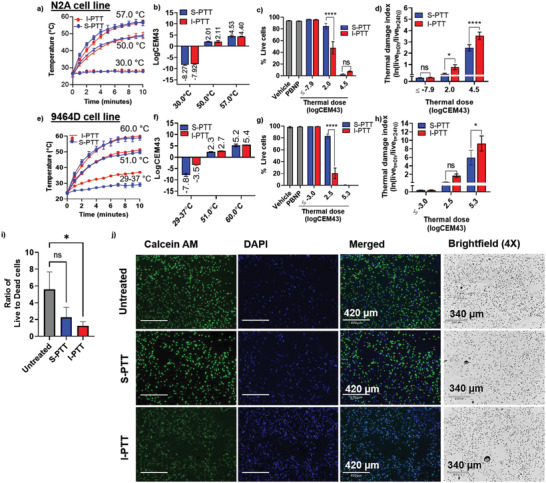
I‐PTT triggers increased cytotoxicity and thermal damage in targeted tumor cells compared to S‐PTT at equivalent thermal doses. N2A and 9464D neuroblastoma cells were suspended in PBS, in the presence or absence of PBNPs, and treated with S‐PTT (blue) and I‐PTT (red). a) N2A cells were heated to final temperatures of 30 °C (no PBNPs), and 50 and 57 °C after 10 min with PBNPs (0.1 mg mL^–1^) at varying laser powers. Temperatures were recorded every minute using a thermal camera. b) The thermal doses (logCEM43) were calculated from the temperature versus time curves in panel (a) for S‐PTT (blue) and I‐PTT (red). c) After treatment, N2A cells were plated and incubated at 37 °C for 24 h, after which the cells were analyzed by flow cytometry to determine N2A cell viability. d) Thermal damage indices for N2A cells were calculated using Equation (1). e) 9464D cells were heated to final temperatures of 29–37 °C (no PBNPs), and 51 and 60 °C for 10 min with PBNPs (0.15 mg mL^–1^) at varying laser powers. Temperatures were recorded every minute using a thermal camera. f) The thermal doses (logCEM43) were calculated form the temperature versus time curves in panel (e) for S‐PTT (blue) and I‐PTT (red). g) After treatment, 9464D cells were plated and incubated at 37 °C for 24 h, after which the cells were analyzed by flow cytometry to determine 9464D cell viability. h) Thermal damage indices for 9464D cells were calculated using Equation (1). i) Ratio of calcein AM‐ to DAPI‐positive 9464D cells to calculate the ratio of live to dead cells. j) Fluorescent and brightfield microscopy images of 100 µm sections stained with calcein AM for live cells and DAPI for dead cells 4 h after I‐PTT or S‐PTT. Values represent means ± st. dev.; ns: not significant, *p < 0.05, **p < 0.01, ***p < 0.001, ****p < 0.0001 (ANOVA, *t*‐test).

To determine the effect of I‐PTT on triggering cytotoxicity and thermal damage, we administered I‐PTT and S‐PTT to two neuroblastoma tumor lines, N2A and 9464D, in vitro using equivalent final temperatures and thermal doses (**Figure** **3**). The temperatures and thermal doses were kept consistent between I‐PTT and S‐PTT to compare the cytotoxic effects generated from the different laser light delivery modes using each PTT modality rather than from the differences in the temperatures attained by each PTT modality. N2A cells were heated to final temperatures of 50 and 57 °C with both I‐PTT and S‐PTT by varying the laser power, while cells treated with I‐PTT or S‐PTT without PBNPs heated to 30 °C (Figure 3a). The resulting thermal doses (logCEM43) achieved for both I‐PTT and S‐PTT were ≈≤ −7.9, 2.0, and 4.5 corresponding to the final temperatures of 30, 50, and 57 °C, respectively (Figure 3b). To determine whether the mode of delivery of PTT (I‐PTT or S‐PTT) affected PTT‐induced cell death, the cell viability was quantitated and used to calculate the thermal damage index, a parameter first described by Viglianti et al., which quantitates the logarithm of the ratio of undamaged cells prior to treatment (time *t* = 0 h) to that measured post‐treatment (*t* = 24 h; Equation (1))^[^ ^47^ ^]^

(1) $$\mathrm{Thermal}\;\mathrm{damage}\;\mathrm{index}\;=\;\ln\left(\frac{\mathrm{live}\left(t\;=\;0\right)}{\mathrm{live}\;\left(t\;=\;24\right)}\right)$$

As expected, I‐PTT significantly decreased N2A cell viability to 47.7% at a thermal dose of 2.0 compared to 84.7% viability observed after S‐PTT at the equivalent thermal dose (Figure 3c and Figure S4, Supporting Information). At the higher thermal dose of 4.5, tumor cell viability decreased to 10% viability with negligible differences between I‐PTT and S‐PTT. The viability was used to determine the number of live cells 24 h after treatment and applied to Equation (1) to calculate the thermal damage index. I‐PTT generated thermal damage indices of 0.8 and 3.5 at the respective thermal doses of 2.0 and 4.5, which were both significantly higher than the thermal damage indices 0.16 and 2.5 observed for S‐PTT for the same corresponding thermal doses (Figure 3d).

To verify these findings, 9464D cells were treated with I‐PTT and S‐PTT. In the absence of PBNPs, 9464D cells heated to 29–37 °C, while in the presence of PBNPs, the cells heated to 51 and 60 °C by varying the laser power (Figure 3e). These final temperatures corresponded to thermal doses of ≤ −3.5, 2.5, and 5.3 for both I‐PTT and S‐PTT (Figure 3f). Similar to the studies with N2A cells, we observed that 9464D viability significantly decreased to 20% with I‐PTT compared to the 83% viability observed with S‐PTT at the low thermal dose of ≈2.5. The reduction in cell viability was similar at the higher thermal dose of 5.3 for both PTT modes (Figure 3g and Figure S5, Supporting Information). However, I‐PTT treatment yielded higher thermal damage indices of 1.7 and 9.2 compared to the thermal damage indices of 0.2 and 5.9 observed with S‐PTT when compared at the thermal doses of 2.5 and 5.3, respectively (Figure 3h). To determine the cell death in gel phantoms, 9464D cells were embedded in agar gels and then treated with I‐PTT and S‐PTT. Again, when the cells embedded in agar gels were heated to similar thermal doses (Figure S6, Supporting Information), the ratio of calcein AM‐stained live cells to 4′,6‐diamidino‐2‐phenylindole (DAPI)‐stained dead cells, calculated using the analyze particles function on Image J, was 1.25 with I‐PTT compared to 2.26 with S‐PTT and 5.59 with untreated embedded cells (Figure 3i,j). Together, the results indicate I‐PTT triggers increased cytotoxicity and thermal damage in targeted tumor cells, compared to S‐PTT. Further, these effects are more evident at lower thermal doses (≈2.0–2.5), suggesting that I‐PTT can reduce the thermal dose for treatment while continuing to achieve effective thermal ablation.

### 2.3 I‐PTT Induces Favorable Markers of Immunogenic Cell Death at a Reduced Thermal Dose

**Figure 4:**
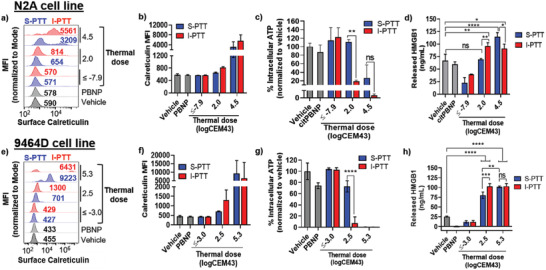
I‐PTT induces favorable correlates of ICD at a lower thermal dose compared to S‐PTT. N2A and 9464D neuroblastoma cells that were suspended in PBS in the presence or absence of PBNPs and treated with S‐PTT (blue) and I‐PTT (red) were assessed for the expression of ICD correlates. Expression of surface calreticulin was measured on N2A cells 24 h after treatment by flow cytometry, represented as a) histograms and the b) corresponding MFI bar graphs. c) Intracellular ATP levels were measured 24 h after treatment by a luminescence‐based assay to indirectly assess the ATP released by treated N2A cells. d) The concentration of intracellular HMGB1 (ng mL^–1^) released in supernatants of N2A cells at 24 h after treatment, as measured by an HMGB1 ELISA. Expression of surface calreticulin was measured on 9464D cells 24 h after treatment as measured by flow cytometry, represented as e) histograms and the f) corresponding MFI bar graphs. g) Intracellular ATP levels were measured 24 h after treatment by a luminescent‐based assay to indirectly assess the ATP released by treated 9464D cells. h) The concentration of intracellular HMGB1 (ng mL^–1^) in supernatants of 9464D cells at 24 h after treatment, as measured by an HMGB1 ELISA. Values represent means ± st. dev. *n* = 3–4 per group; ns: not significant, *p < 0.05, **p < 0.01, ***p < 0.001, ****p < 0.0001 (ANOVA).

To assess if I‐PTT confers an advantage over S‐PTT in generating ICD in treated tumor cells, we administered both I‐PTT and S‐PTT to N2A and 9464D neuroblastoma cells over a range of thermal doses. We measured the effects of I‐PTT and S‐PTT on published, consensus ICD correlates calreticulin, adenosine triphosphate (ATP), and HMGB1 as a function of thermal dose.^[^ ^48^ ^]^ In the study with N2A tumor cells, I‐PTT induced the higher surface expression of calreticulin compared to S‐PTT, with median fluorescence intensity (MFI) levels increasing from 814 to 5561 with I‐PTT, while S‐PTT generated MFIs of 654 and 3209 at thermal dose of 2.0 and 4.5, respectively (**Figure** **4**a,b and Figure S7, Supporting Information), although this was not statistically significant. Meanwhile, N2A cells heated at the lowest thermal dose ≤ −7.9 had similar surface calreticulin expression as vehicle and PBNP nonheated cells, indicating that PBNPs are mediating the light to heat conversion, and that expression of calreticulin is associated with nanoparticle‐based photothermal heating. When assessing the levels of intracellular ATP at the thermal dose of 2.0 in the N2A‐PTT treated cells, I‐PTT significantly decreased the levels of intracellular ATP level to 40.5% compared to the 82.7% observed with S‐PTT (Figure 4c). At a thermal dose of 4.5, intracellular ATP further decreased to ≈1% with both PTT strategies, with no significant difference between I‐PTT and S‐PTT. The last marker assessed was intracellular HMGB1 released into the supernatant after PTT. S‐PTT at a thermal dose of 2.0 generated release of 68.9 ng mL^–1^ HMGB1 into the supernatant, similar to vehicle and PBNP samples, but significantly less than the 96.1 ng mL^–1^ HMGB1 released after I‐PTT treatment at the same thermal dose (Figure 4d). At the higher thermal dose of 4.5, the released HMGB1 concentration with S‐PTT increased to 114.4 ng mL^–1^ compared to the lower levels observed at the thermal dose of 2.0. By comparison, at the thermal dose 4.5, I‐PTT generated a 91.1 ng mL^−1^ HMGB1 release similar to that observed at a thermal dose of 2.0, suggesting that with I‐PTT, the maximum release of HMGB1 was achieved at the low thermal dose of 2.0 and maintained at the higher thermal dose of 4.5. These data suggest that in N2A cells, while increased ICD correlates were observed at a thermal dose of 4.5 with either PTT strategy, the I‐PTT strategy had an enhanced effect over S‐PTT in inducing favorable ICD correlates at the lower thermal dose.

Similar to the N2A study, the 9464D PTT‐treated cells exhibited increased surface calreticulin MFIs increasing from 1300 to 6431 for I‐PTT, while S‐PTT generated MFIs of 701 and 9223 at thermal dose of 2.5 and 5.3, although the differences between I‐PTT and S‐PTT were not statistically significant (Figure 4e,f and Figure S8, Supporting Information). At the thermal dose of 2.5, 9464D I‐PTT treated cells also showed a dramatic decrease in intracellular ATP levels (7.4%) compared to 73.1% observed with S‐PTT. Yet, at a thermal dose of 5.3, intracellular ATP further decreased to less than 1% with both PTT strategies, with no significant differences between I‐PTT and S‐PTT, similar to the trend we had observed with N2A I‐PTT treated cells in Figure 4c (Figure 4g). With released HMGB1, I‐PTT induced a higher concentration of released HMGB1 (102.6 ng mL^–1^) at the thermal dose of 2.5, and it was maintained when a thermal dose of 5.3 was applied (Figure 4h), similar to observations from N2A cells in Figure 4d. Treatment with S‐PTT reached the highest released HMGB1 concentration of 101.5 ng mL^–1^ at a thermal dose of 5.3, while it only demonstrated a release of 79.9 ng mL^–1^ HMGB1 at the lower thermal dose of 2.5, which was significantly lower than I‐PTT at the same thermal dose. Thus, our data with I‐PTT‐treated 9464D cells demonstrate similar trends as the I‐PTT‐treated N2A cells when assessing the induction of ICD at a low thermal dose of 2.0/2.5 where I‐PTT had an enhanced effect over S‐PTT. At the higher thermal doses of 4.5/5.3, both I‐PTT and S‐PTT could elicit equivalent ICD correlates. Overall, these findings demonstrate that the improved efficiency of heating provided by I‐PTT improves the immunogenicity of the dying tumor cells when compared with S‐PTT and this improvement is more pronounced at a lower thermal dose.

### 2.4 I‐PTT Expands the Treatment Zone at or above the Thermal Breakpoint In Vivo

**Figure 5:**
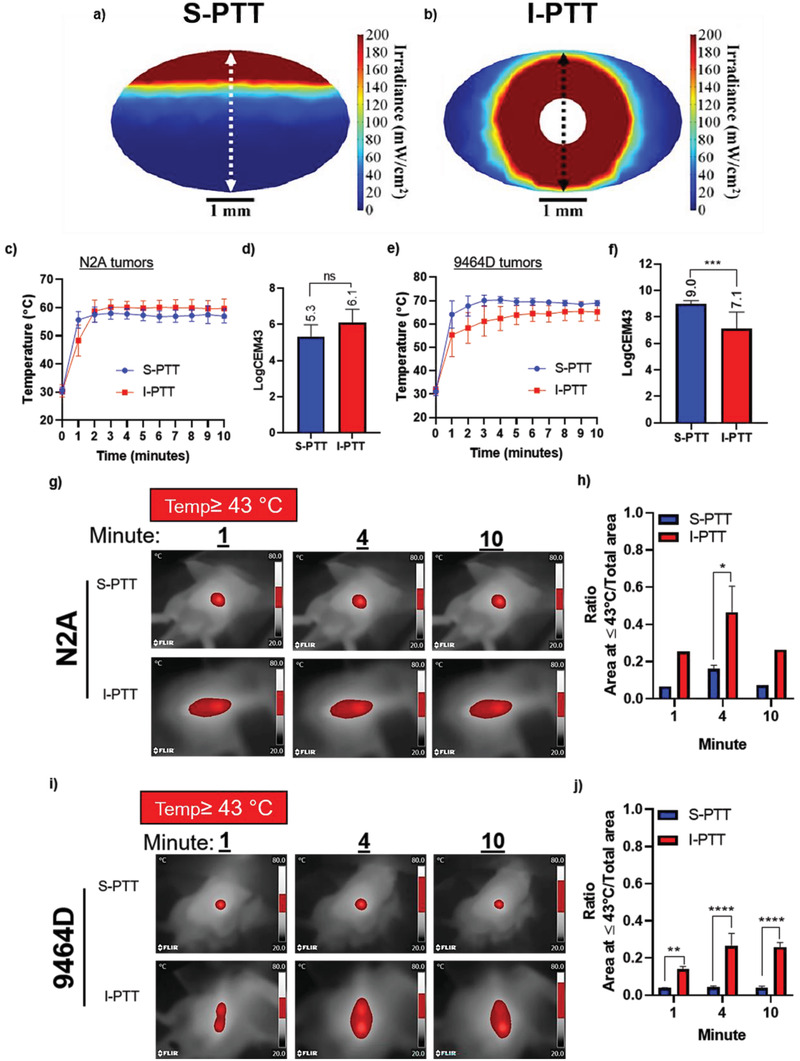
I‐PTT expands the treatment zone at or above the thermal breakpoint in vivo. Simulated cross‐sectional irradiance distribution within a 5 mm by 5 mm by 3 mm (length x width x depth) tumor for a) S‐PTT and b) I‐PTT in the range of 0–200 mW cm^–2^. N2A and 9464D tumor bearing mice were administered PBNPs (2.5 mg kg^–1^) via i.t. injection and tumors were then exposed to NIR laser irradiation for PTT. c) N2A tumors were heated to a superficial temperature of ≈60 °C for both I‐PTT and S‐PTT, as measured by a thermal camera. d) The thermal doses (logCEM43) achieved using both PTT configurations calculated from the temperatures versus time curves in panel (a) for N2A tumors. e) 9464D tumors were heated to superficial temperatures of at least 60 °C for both treatments, as measured by a thermal camera. f) The thermal doses (logCEM43) for both PTT configurations calculated from panel (c) for 9464D tumors. g) Representative thermal images of N2A tumor‐bearing mice treated with S‐PTT (top) or I‐PTT diffuser (bottom), showing the area heating at or above 43 °C (red), the tissue thermal breakpoint, at minutes 1, 4, and 10 of PTT. h) The quantitated ratio of the area at or above 43 °C to the fixed total area for S‐PTT and I‐PTT treatments in the N2A model. i) Representative thermal images of 9464D tumor‐bearing mice treated with S‐PTT (top) or I‐PTT diffuser (bottom), showing the area heating at or above 43 °C (red) during minutes 1, 4, and 10 of PTT. j) The quantitated ratio of the area at or above 43 °C to the fixed total area for S‐PTT and I‐PTT treatments in the 9464D model. Values represent means ± st. dev. *n* = 10 per group for panels (a)–(d). *n* = 3 for panels (e)–(h); ns: not significant, *p < 0.05, **p < 0.01, ***p < 0.001, ****p < 0.0001 (ANOVA).

Building on the heating advantages that I‐PTT demonstrated in vitro, we conducted studies in vivo to measure heat distributions during I‐PTT in mice‐bearing single N2A or 9464D tumors. First, to examine the differences between S‐PTT and I‐PTT in tissue heating, we conducted finite element method (FEM) simulations. Using an FEM approach, our coauthors have previously demonstrated that tissue heating is directly proportional to the light propagation within the tissue.^[^ ^49^, ^50^, ^51^ ^]^ For the S‐PTT and I‐PTT simulations, the tumor size was assumed to be 5 mm by 5 mm in length and width, with a tumor depth of 3 mm (Figure S8a,b, Supporting Information), and the optical properties are described in Table S1, Supporting Information. The FEM simulations demonstrated that the irradiance for S‐PTT is mainly localized at the surface of the tumor with minimal irradiance in the center of the tumor (**Figure** **5**a). By contrast for I‐PTT, the irradiance covers a greater area in the center of the tumor (Figure 5b).

To compare the therapeutic effects of I‐PTT with S‐PTT in the N2A tumor model, we adjusted the laser powers for each PTT modality to attain treatment temperatures of 60 °C with each modality during the 10 min treatment period. Previous studies in our group with superficially administered PBNP‐PTT of the N2A tumors at temperatures of 60 °C generated an optimal thermal dose to completely ablate tumor with low tumor recurrence.^[^ ^9^, ^10^ ^]^ Therefore, to determine if interstitially administered PBNP‐PTT could similarly generate complete tumor ablation, we administered I‐PTT at 60 °C, as measured by a thermal camera (Figure 5c). The corresponding thermal doses were 6.1 or 5.3 for I‐PTT and S‐PTT, respectively (Figure 5d). In the 9464D model, we varied the laser powers for both PTT modalities to administer the treatments to at least 60 °C. Previous preliminary studies with PBNP‐PTT of 9464D tumors (not published) suggested that superficially administered PBNP‐PTT at temperatures <60 °C generated a sub‐optimal thermal dose for tumor ablation. Limitations with the lack of cooling for the interstitial fibers restricted the I‐PTT treatment temperature to around 60 °C, and thus, to conduct a comparative study of I‐PTT and S‐PTT in the 9464D model, tumors were administered PBNP‐PTT with either PTT modality at treatment temperatures of at least 60 °C (Figure 5e). The corresponding thermal doses were 7.1 and 9.0 for I‐PTT and S‐PTT, respectively (Figure 5f). Interestingly, we observed that the I‐PTT treatments heated a greater area of the flank of N2A tumor‐bearing mice at or above 43 °C the thermal breakpoint of tissue^[^ ^52^ ^]^ (red; Figure 5g), which was a 2.8‐ to 3.8‐fold greater area than that for S‐PTT at minutes 1, 4, and 10 (Figure 5h). We observed a similar trend in 9464D tumor‐bearing mice undergoing I‐PTT, with a 3.6‐ to 6.8‐fold increase of the area of the flank attaining temperatures at or above 43 °C at similar time‐points (Figure 5i,j) compared to S‐PTT. Although the area at or above the thermal breakpoint of 43 °C includes tissue/skin that may not present a palpable tumor, providing this treatment at areas surrounding the tumor margin could improve the treatment's efficacy to eliminate tumor cells at the margins that are not palpable.^[^ ^53^ ^]^

Our data suggest that I‐PTT can be administered at thermal doses comparable to S‐PTT in murine neuroblastoma models. Further, I‐PTT heats a larger area at or above the tissue thermal breakpoint.

### 2.5 I‐PTT Generates Significantly Higher Tumor Regression, Long‐Term Survival, and Protection against Tumor Rechallenge in Syngeneic Models of Neuroblastoma Compared to S‐PTT

**Figure 6:**
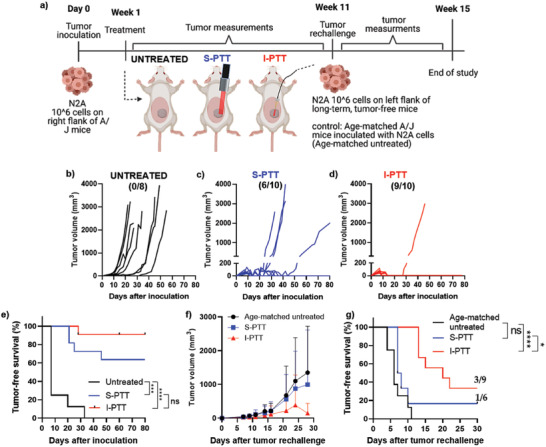
I‐PTT generates significantly higher tumor regression, long‐term survival, and protection against tumor rechallenge in the N2A model of neuroblastoma. a) Study schematic showing groups and treatments. A single subcutaneous N2A tumor was inoculated on the right flank of A/J mice. Around week 1, tumors were either left untreated, or were administered S‐PTT or I‐PTT after i.t. injection of PBNPs (2.5 mg kg^–1^). Tumor sizes were monitored daily. Long‐term surviving, tumor‐free mice were rechallenged on Week 11 (Day 80) to assess protection against tumor recurrence. Tumor growth was monitored daily until Day 30 after tumor rechallenge (Day 110 overall). b–d) Individual tumor growth curves of untreated tumors (black), and S‐PTT (blue) and I‐PTT (red) treated tumors. Numbers in parentheses indicate the number of tumor‐free mice by Day 80. e) Tumor‐free survival rate of mice as of Day 80 after either PTT treatment compared to control, with each event indicating the presence of a tumor. f) Average volume of rechallenge tumors. g) Tumor‐free survival rate of mice after N2A tumor rechallenge. *n* = 8–10 per group for panels (b)–(e). *n* = 6–9 per group for panel (f)–(h); log‐rank test; ns: not significant, *p < 0.05, **p < 0.01, ***p < 0.001, ****p < 0.0001 (Log‐rank).

**Figure 7:**
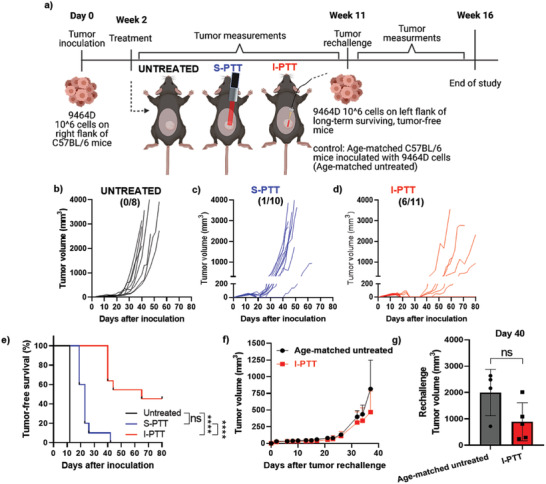
I‐PTT generates significantly higher tumor‐free survival after treatment in the 9464D model of neuroblastoma. a) Study schematic diagram showing groups and treatments. A single subcutaneous 9464D tumor was inoculated on the right flank of C57BL/6 mice. After 13–16 days, tumors were either left untreated, or administered S‐PTT or I‐PTT after i.t. injections of PBNPs (2.5 mg kg^–1^). Tumor sizes were monitored daily. Long‐term surviving, tumor‐free mice were rechallenged on week 11 (Day 80) to assess protection against tumor recurrence. Tumor growth was monitored daily until Day 30 after tumor rechallenge. b–d) Individual tumor growth curves of untreated tumors (black), and S‐PTT (blue) and I‐PTT (red) treated tumors. Numbers in parentheses indicate the number of tumor‐free mice by Day 80. e) Tumor‐free survival rate of mice by Day 80, after treatment. f) Average volume of rechallenge tumors. g) Average volume of rechallenge tumors at Day 40 after tumor extraction. *n* = 8–10 per group for panels (b)–(e). *n* = 4–5 per group for panel (f)–(h); log‐rank test; ns: not significant, *p < 0.05, **p < 0.01, ***p < 0.001, ****p < 0.0001 (Log‐rank).

Complementing the thermal analysis studies of I‐PTT in vivo, we sought to determine the therapeutic effect of the I‐PTT treatment by evaluating tumor progression and tumor‐free survival in response to I‐PTT, S‐PTT, and control treatments in the N2A (**Figure** **6**) and 9464D (**Figure** **7**) murine models of neuroblastoma.

In the N2A tumor model (study plan detailed in Figure 6a), tumors were treated once the volume reached between 44 and 60 mm^3^. These tumor sizes allowed for a fair comparison of S‐PTT and I‐PTT as the superficial laser spot size is 40 mm^2^. Upon treatment initiation with either I‐PTT or S‐PTT, the tumors were monitored daily for tumor progression. As expected, upon treatment with either S‐PTT or I‐PTT, both treated and control untreated tumors initially regressed in the days after treatment while tumors on the untreated mice progressed (Figure 6b–d). However, by Day 20–30 after inoculation (Day 13–23 after treatment), 3 of 10 S‐PTT‐treated mice (blue) and 1 of 10 I‐PTT‐treated mice (red; Figure 6b–d) exhibited tumor recurrence. By the time of tumor rechallenge on week 11, only 6 of 10 S‐PTT‐treated mice were tumor‐free while I‐PTT treatment resulted in 9 of 10 tumor‐free mice, with these mice from both treatment groups remaining disease‐free through week 11. This resulted in tumor‐free survival improving to 90% with I‐PTT compared to the 60% observed with S‐PTT (Figure 6e). Thus, I‐PTT effectively ablated N2A tumors and decreased tumor recurrence to a higher extent than S‐PTT.

Next, we evaluated whether the observed antitumor response would provide long‐lasting protection against tumor rechallenge as a measure of immunological memory post‐treatment. In long‐term, tumor‐free surviving mice from the S‐PTT and I‐PTT groups, we observed improved protection against tumor rechallenge in the I‐PTT treatment groups, as indicated by the slower growth of rechallenge tumors compared to S‐PTT and age‐matched untreated mice (Figure 6f and Figure S10, Supporting Information). Additionally, I‐PTT delayed the onset of the rechallenge tumor to Day 13 after tumor rechallenge while S‐PTT‐treated mice exhibited tumor recurrence by Day 7 after tumor rechallenge (Figure 6g). By Day 30 after tumor rechallenge, 1 of 6 (16.7%) rechallenged mice in the S‐PTT group survived the tumor rechallenge without any tumor progression. Remarkably, 3 of 9 (33.3%) rechallenged mice in the I‐PTT group exhibited tumor‐free survival, significantly improving the tumor‐free survival compared to that observed with the S‐PTT group.

To verify the findings demonstrating the potency of I‐PTT in vivo to thermally ablate established N2A tumors, we conducted similar studies in the 9464D neuroblastoma tumors model. Similar to the N2A model, in the 9464D tumor model (study plan detailed in Figure 7a), tumors were treated at volume of 44 to 60 mm^3^. Upon treatment with either I‐PTT or S‐PTT, the tumors were monitored daily for tumor progression along with control untreated tumors. Unlike the N2A model, the 9464D tumors treated with S‐PTT demonstrated a higher tumor recurrence rate with 8 of 10 mice demonstrating recurrence by Day 24 (1 week after treatment), and the tumor growth was similar to untreated tumors (Figure 7b,c). By Day 40, 9 of 10 mice demonstrated tumor recurrence in the S‐PTT group. By contrast, all 11 I‐PTT‐treated mice were tumor‐free at Day 24 (1 week after treatment). By Day 40 after inoculation, 5 of 11 I‐PTT‐treated mice demonstrated tumor recurrence (Figure 7d). As a result, I‐PTT significantly increased the tumor‐free survival to 45%, while 0% of S‐PTT‐treated mice were tumor‐free by Day 80 (Figure 7e). On Day 80, 6 out of 11 I‐PTT mice were tumor‐free and thus included in the tumor rechallenge study.

Similarly to our previous study, by Day 80, the tumor‐free mice in the I‐PTT group were rechallenged with 9464D cells to assess the antitumor protection conferred by I‐PTT. While rechallenge tumors were observed for both I‐PTT and age‐matched untreated mice (Figure S11, Supporting Information), after Day 26, the average tumor volume on I‐PTT‐treated mice was smaller than that on treatment naive tumor‐bearing mice (Figure 7f). On Day 40, tumors were removed from mice and measured ex vivo (Figure S12, Supporting Information). Overall, I‐PTT reduced the tumor volume by 2.25‐fold, decreasing the final average tumor volume to 887.6 mm^3^ compared to the average tumor volume of 1999.3 mm^3^ on naive tumor‐bearing mice (Figure 7g), although this difference was not significant. Finally, since PBNPs are highly stable when synthesized and stored, we conducted studies to address the potential long‐term toxicity of PBNPs. We observed that PBNPs do not induce hemolysis (Figure S13, Supporting Information) when suspended in blood of tumor‐free mice. Further, PBNPs injected subcutaneously into 50 weeks old healthy mice at 2.5 mg kg^−1^, equivalent to our in vivo studies above, did not cause alanine aminotransferase (ALT) and aspartate aminotransferase (AST) liver enzyme levels to change relative to untreated healthy mice not injected with PBNPs (Figure S14a,b, Supporting Information). Additionally, several organs harvested from PBNP‐injected mice at Day 36 after injection appeared visually similar to untreated healthy mice (Figure S14c, Supporting Information). These studies complement previously published studies from our group including PBNPs only arm in vivo for both neuroblastoma tumor models where it was observed that while PBNPs alone may slow tumor progression in the 9464D model, there were no significant differences in overall survival in this PBNPs alone treatment group relative to untreated, control tumor‐bearing mice for either model. Therefore, this group was not included in our study.^[^ ^9^, ^12^ ^]^

Taken together, these data suggest that I‐PTT in murine models of neuroblastoma can 1) generate complete ablation of established tumors and improve tumor‐free survival after tumor rechallenge when treatment is administered at optimal thermal doses, while 2) at sub‐optimal thermal doses, I‐PTT is more effective in achieving complete tumor ablation and thus reduces the tumor recurrence rate after treatment.

### 2.6 I‐PTT Yields a Comparable Therapeutic Benefit as S‐PTT in a Two‐Tumor 9464D Model of Neuroblastoma but at a Decreased Thermal Dose

**Figure 8:**
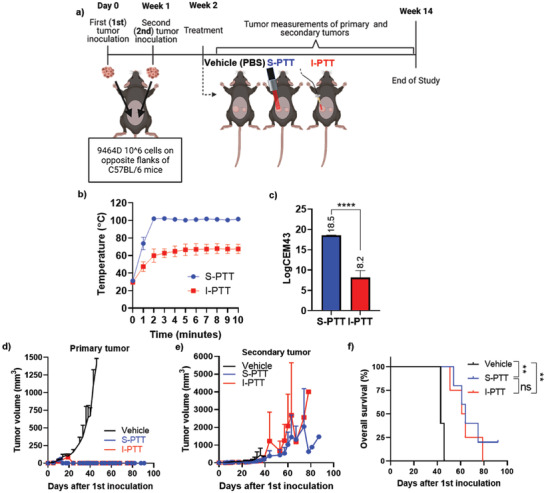
I‐PTT yields a comparable therapeutic benefit as S‐PTT in a two‐tumor 9464D model of neuroblastoma but at a decreased thermal dose. a) Study schematic showing groups and treatments. A single subcutaneous 9464D tumor was inoculated on the right flank of C57BL/6 mice on Day 0, and a secondary tumor was inoculated on the left flank on Day 7. 12 to 19 days after the first tumor inoculation, the primary tumor was either left untreated, or was administered S‐PTT or I‐PTT after i.t. injection of PBNPs (2.5 mg kg^–1^). Tumor sizes for both the primary and secondary tumors were monitored daily. The overall survival was monitored to assess systemic antitumor effects. b) For the PTT treatments, mice were treated for 10 min at ≈100 °C for S‐PTT (blue) and ≈60 °C for I‐PTT (red). c) The thermal doses (logCEM43) attained with S‐PTT (blue) and I‐PTT (red) treatment. d) Average tumor volumes corresponding to the primary tumors of vehicle‐treated, S‐PTT‐, or I‐PTT‐treated mice. e) Average tumor volumes corresponding to the secondary tumors of vehicle‐treated, S‐PTT‐, and I‐PTT‐treated mice. f) Overall survival rate of mice by Day 100, after the first tumor inoculation. *n* = 4–5 per group. log‐rank test; ns: not significant, *p < 0.05, **p < 0.01, ***p < 0.001, ****p < 0.0001 (Log‐rank).

To understand the systemic antitumor effects of I‐PTT, we conducted a study using a metachronous 9464D tumor model with two tumors on opposing flanks inoculated 1 week apart (**Figure** **8**a). Once the first tumor reached a volume of ≈70 mm^3^, this tumor was i.t. injected with PBNPs and treated with either PTT strategy (S‐PTT or I‐PTT) and hereafter mentioned as primary tumor. For vehicle‐treated mice, the primary tumors were injected with phosphate‐buffered serum (PBS) and monitored for tumor progression.

I‐PTT was applied at a tumor superficial temperature of 65 °C, while S‐PTT was applied at 100 °C (Figure 8b), leading to thermal doses of 8.2 and 18.5 logCEM43 for I‐PTT and S‐PTT, respectively (Figure 8c). I‐PTT was administered at a significantly lower thermal dose similar to Figure 7 as it was observed that complete tumor ablation with I‐PTT can be attained at this thermal dose. In addition, lower temperatures prevent damage to the diffuser fiber in the absence of a cooling system. By contrast, S‐PTT with PBNPs had previously been observed to elicit complete tumor ablation in 9464D tumors only at thermal doses at least twofold higher.^[^ ^12^ ^]^ Upon PTT treatment, the high thermal dose of S‐PTT was able to completely ablate the primary tumor without recurrence in 5 out of 5 mice‐bearing 9464D tumors, meanwhile I‐PTT successfully ablated tumors without recurrence in 4 out of 4 mice at the lower thermal dose (Figure 8d). By comparison, the primary tumor of vehicle‐treated mice grew rapidly, reaching an end‐point tumor volume by Day 40. To assess if the treatments generated an abscopal effect to elicit an antitumor response on the untreated tumor, we observed the tumor growth of the secondary, untreated tumor. Both S‐PTT and I‐PTT were not able to induce a strong enough antitumor response to completely regress the secondary, untreated tumor, with active tumor progression observed in mice treated with both modes of PTT (Figure 8e). Secondary untreated tumors in S‐PTT‐treated mice progressed slightly slower than secondary, untreated tumors on I‐PTT‐treated mice, yet this was found not be significant. We also demonstrated that S‐PTT and I‐PTT achieved statistically comparable overall survival rates (Figure 8f).

Overall, this 9464D metachronous model study demonstrated that while I‐PTT could ablate harder‐to‐treat 9464D tumors and generate a similar antitumor abscopal effect on secondary tumors as S‐PTT, these effects were achieved at a much lower thermal dose than S‐PTT, further highlighting the efficient heating that can be attained with I‐PTT.

## 3 Discussion

In this study, we described the use of interstitial PTT (I‐PTT), which comprised of i.t. injected PBNPs illuminated by an interstitial laser delivered using an optical fiber, in the N2A and 9464D models of neuroblastoma. We implemented the delivery of light using an optical fiber fitted with a cylindrical diffuser to target a treatment area (in vitro) or the tumor (in vivo). This mode delivers light from the inside‐out as compared to superficial external beam illumination typically explored in most PTT‐based studies (Figure 1). For light‐based treatments such as interstitial PDT, light is typically delivered by optical fibers with a flat‐cut end or a terminal diffuser. Shafirstein et al. modeled the light from the flat‐cut end fiber to illuminate a diffused ball within a tumor, while in a cylindrical diffuser, the light is emitted radially along the diffuser length to illuminate a diffuser cylinder within the tumor and thus a larger volume in the tumor compared to a flat‐cut end fiber.^[^ ^54^ ^]^ Confirming these expected advantages, in our studies we observed that I‐PTT Diffuser yielded both an improved efficiency of heating and heat distribution at the same output laser power as compared to I‐PTT Flat‐cut and S‐PTT (Figure 2). These observations suggest that the laser light emitted radially from the diffuser could reach more PBNPs than I‐PTT Flat‐cut or S‐PTT, allowing more PBNPs to absorb laser light for light‐to‐heat conversion, leading to the higher temperatures. Our findings are consistent with observations in PDT where cylindrical diffusers have been shown to be more effective in delivering laser light and energy per fiber to the targeted tumors than flat‐cut fibers.^[^ ^55^ ^]^

Both I‐PTT strategies exhibited heating of a target treatment area from the inside‐out while the heating with S‐PTT was localized at the top of the solution (Figure 2e). This observation visually depicts the main limitation with using external/superficial‐based approaches for deep‐seated tumors. Thus, if the fibers can be placed directly into the tumor, a greater area of the tumor can be treated, allowing for the resulting heating to be more effective than S‐PTT. This suggests that I‐PTT (using a cylindrical diffuser) could improve the treatment efficacy not only for deep‐seated tumors, but also for accessible superficial lesions. Limited depth of penetration continues to be a limitation in light‐based therapies for skin cancers.^[^ ^56^, ^57^, ^58^ ^]^ Therefore, the introduction of interstitial light delivery could potentially improve treatment efficacy for these applications. For both murine neuroblastoma tumor lines in vitro, I‐PTT yielded tumor cell viability that was significantly reduced compared to S‐PTT at the low thermal dose, but S‐PTT could kill the tumor cells comparably to I‐PTT at the higher thermal dose (Figure 3). However, in terms of inducing thermal damage, I‐PTT consistently generated a higher damage index at all thermal doses tested, indicating its advantage in killing more tumor cells compared with S‐PTT.

Our group and others have demonstrated that PTT as a monotherapy leads to effective short‐term tumor ablation and control. However, this is commonly followed by tumor recurrence, especially in aggressive cancer models.^[^ ^12^, ^15^, ^16^, ^39^ ^]^ In tumors that were heated to similar superficial temperatures, I‐PTT yielded a larger coverage area of treatment that encompassed tissue beyond the tumor volume, which may have potentially extended the treatment benefit to destroy the tumor margins (Figure 5). The tumor margin has been implicated as the source for tumor recurrence in many ablative therapies, particularly when the ablation/treatment zones do not extend far from the target tumor area.^[^ ^53^, ^59^, ^60^, ^61^ ^]^ With PTT, if the thermal ablation area excludes tumor margins, tumor recurrence occurs rapidly.^[^ ^62^ ^]^ In vivo, when I‐PTT was administered to mice‐bearing neuroblastoma tumors at equivalent thermal doses to S‐PTT (Figures 6 and 7), we observed a significantly reduced rate of tumor recurrence as compared to S‐PTT, suggesting the efficacy of I‐PTT is not only eradicating tumor but also controlling their margins to reduce the incidence of recurrence. A critical aspect of our I‐PTT approach is the use of PBNPs. These nanoparticles retained their size distribution and surface charge over time without aggregating, as indicated by the consistent PDI measured over 2 years post‐synthesis. Since PBNPs are highly stable when synthesized and stored, one concern relates to the long‐term toxicity of these nanoparticles in vivo. In Balakrishnan et al., we demonstrated that PBNP‐PTT‐treated melanoma tumor‐bearing mice exhibited similar liver histology as untreated tumor‐bearing mice, indicating minimal short‐term toxicity of PBNPs after PTT.^[^ ^8^ ^]^ Another group demonstrated that intravenous PBNPs have acute, short‐term hepatotoxicity within the first 24 h of injection that return to normal levels after 3 to 7 days,^[^ ^33^ ^]^ indicating that low toxicity is expected from i.t. injected PBNPs in our study. Compared to this study, we utilized a PBNP dose that was 3.2‐fold lower (2.5 mg kg^–1^ in our study vs 8.0 mg kg^–1^). Our studies demonstrated that the PBNPs did not induce lysis of red blood cells after short‐term exposure of whole blood to the PBNPs ex vivo (Figure S13, Supporting Information). Complementary to our earlier studies in tumor‐bearing mice, in this study we also observed similar ALT and AST levels between PBNP‐injected and untreated healthy mice (Figure S14, Supporting Information), further supporting the minimal toxicity expected with PBNPs after long‐term exposure in vivo. Additionally, as described earlier, Prussian blue is already an FDA‐approved material used to eliminate radioactive isotopes.^[^ ^31^, ^32^ ^]^ Therefore, the PBNPs used in this study are suitable for further preclinical development and potential clinical translation.

The i.t. administration of PBNPs facilitates PTT to be administered locally at the tumor site and margins to minimize off‐target heating. This mitigates concerns related to rapid clearance, low tumor localization, and retention commonly observed with systemically administered nanoparticles. However, while i.t. delivery of drugs is currently being clinically evaluated and has been observed to have high efficacy against tumors in some settings,^[^ ^63^, ^64^ ^]^ one main concern is the feasibility of i.t. injections in solid tumors. Solid tumors present with high interstitial pressure that can affect the distribution, retention, and leakage of the drugs or agents, resulting in variability in delivery per injection. Since i.t. PBNPs are critical in our study to observe temperature increase in the tumor, we overcame any heating differential due to varying i.t. PBNP concentrations by varying the output power of the laser to attain a desired temperature/temperature range in the in vivo studies. However, whether the difference in i.t. PBNP distribution affects the observed heat distribution in the heating zone has not been assessed in this study. To improve i.t. PBNP retention and distribution, future studies should evaluate i.t. administration of PBNPs using infusion needles with sideports coupled to pressure transducers (e.g., ProFusion needles by Cook Regentec).^[^ ^65^ ^]^ This approach provides real‐time pressure measurements during the injection along with delivery of the nanoparticles through its sideports along the needle length to provide a more uniform i.t. nanoparticle delivery compared to the use of a standard needle. Lastly, whether PBNPs induce nanoparticle‐induced endothelial leakiness and consequently provide additional therapeutic benefit of PBNP accumulation within tumors or aid in tumor cell extravasation should be evaluated in future studies.^[^ ^66^, ^67^ ^]^

Despite these promising results, it is important to note that I‐PTT can be further improved by: 1) integrating an imaging system to visualize the fiber placement within the tumor volume, 2) using more than one fiber for treatment based on image‐based pretreatment planning, and 3) accurately measuring temperatures within the tumor volume by a thermal probe. Clinically, MRI or ultrasound guidance is used with LITT, both to monitor fiber placement as well as closely monitor the thermal damage. Additionally, LITT fibers are equipped with cooling systems that help prevent damage to the fibers. As part of the I‐PTT set‐up for the study, the absence of an active fiber tip cooling system limited the maximum superficial temperatures we could assess in our in vivo study (Figure 8). For this initial study, PTT was applied with a single optical fiber, but whether or not illumination of the tumor was sufficient for the tumor volume at treatment time is not known. As we continue to enhance I‐PTT administration, an image‐based pretreatment planning would be essential to determine both the placement of fibers and the number of fibers needed to effectively illuminate and thus treat the tumor by I‐PTT. Lastly, in regards to thermal dosimetry, the thermal camera used for the in vivo studies provided temperature readings at the surface of the tumors. From a superficial temperature stance, the thermal doses achieved for either PTT treatment were similar for studies in Figures 6 and 7, and were drastically different for the study in Figure 8. However, for either PTT modality, temperature readings within the tumor volume using a thermal probe would provide more accurate temperature readings during treatment, and thus more precise calculations of thermal doses were applied at treatment. These important enhancements to I‐PTT are the active focus of studies in our group.

While the thermal effect of PTT is a critical component for its efficacy, the therapeutic effect of PTT is significantly improved when combined with immune adjuvants, with studies demonstrating that PTT can be used to potentiate robust antitumor immune responses.^[^ ^9^, ^10^, ^12^, ^13^, ^15^, ^39^, ^68^, ^69^, ^70^ ^]^ Our studies assessing the ICD correlates of I‐PTT demonstrated that while I‐PTT induced ICD at levels similar to S‐PTT in vitro at the higher thermal doses, the expression of the ICD correlates was substantially enhanced by I‐PTT at the lower thermal dose (Figure 4). In vivo I‐PTT reduced the growth rate of rechallenge tumors in optimally treated murine neuroblastoma, while the systemic tumor protection against tumor rechallenge was limited in treatments with sub‐optimal thermal doses (Figures 6f and 7f). While the rechallenge models are suggestive of immunological memory, further studies into the tumor microenvironment of these rechallenge tumors would be essential to determine whether this effect is immune‐driven and tumor‐specific.^[^ ^68^ ^]^

Finally, in the two‐tumor model of 9464D, we observed similar treatment effects on primary and secondary tumors after I‐PTT and S‐PTT treatments of the primary 9464D tumors (Figure 8). However, in this study, I‐PTT was administered at a significantly lower thermal dose (due to the limitation mentioned earlier because of the absence of a cooling system, and based on the ability to attain tumor ablation at this lower thermal dose) compared to S‐PTT in which we would have expected lower overall survival with I‐PTT. However, the statistically insignificant difference in overall survival observed between I‐PTT and S‐PTT‐treated mice indicates that I‐PTT at the low thermal dose was as therapeutically effective as S‐PTT at the higher thermal dose. Thermal‐based therapies in the clinic are highly monitored to prevent excessive heating in order to minimize damage to nontumor, healthy tissue and consequent adverse hyperthermia‐related events/toxicities.^[^ ^71^, ^72^ ^]^ Consequently, in future studies, we will administer I‐PTT at the lowest thermal dose without compromising treatment efficacy. We also plan to thoroughly characterize the immune response to I‐PTT with the goal of maximizing its efficacy in cancer immunotherapy applications.

## 4 Conclusion

Our study has demonstrated the therapeutic advantage of administering I‐PTT compared to S‐PTT. In vitro, I‐PTT improved the efficiency and distribution of heat within a target treatment area, increased the cytotoxicity and thermal damage in targeted tumor cells at equivalent thermal doses, and induced favorable correlates of ICD at a lower thermal dose compared to S‐PTT. In vivo, I‐PTT expanded the treatment zone at or above the thermal breakpoint, generated significantly higher protection against tumor recurrence and improving long‐term survival in the models of neuroblastoma compared to S‐PTT, and elicited a similar therapeutic response as S‐PTT administered at a significantly higher thermal dose in the metachronous 9464D model. Together, these results indicate that I‐PTT could be a viable option for the treatment of neuroblastoma with minimal invasiveness, and the improvement of the efficacy of PTT when using I‐PTT suggests its further development and translation to treat both superficial and deep‐seated tumors.

## 5 Experimental Section

### Chemical and Biological Reagents

Iron (III) chloride hexahydrate (MW 270.3; FeCl_3_·6H_2_O), potassium hexacyanoferrate (II) trihydrate (MW 422.39; K_4_[Fe(CN)_6_]·3H_2_O), acetone, sodium chloride, and citric acid were all obtained from Sigma‐Aldrich (St. Louis, MO, USA). Eagle's minimum essential medium (EMEM) was purchased from ATCC (Manassas, VA, USA). PBS, Dulbecco's modified Eagle's medium (DMEM), penicillin‐streptomycin, nonessential amino acids, antibiotic‐antimycotic, and *β*‐mercaptoethanol were purchased from Thermo Fisher Scientific (Waltham, MA, USA). Fetal bovine serum (FBS) was purchased from Thermo Fisher Scientific and R&D Systems (Minneapolis, MN, USA). Live/dead zombie violet dye was purchased from BioLegend (San Diego, CA, USA). Fluorescent antibodies against phycoerythrin calreticulin (clone FMC75) were purchased from Abcam (Cambridge, UK). HMGB1 Detection ELISA (enzyme‐linked immunoassay) kit was purchased from Chondrex, Inc. (Woodinville, WA, USA).

### Cells

The murine neuroblastoma cell line Neuro2a (N2A; ATCC) was cultured in EMEM containing 10% FBS (Thermo Fisher Scientific) and 1% penicillin‐streptomycin. The transgenic murine neuroblastoma cell line TH‐MYCN 9464D were provided by Dr. Carol Thiele (Pediatric Oncology Branch, NIH, Bethesda, MD, USA).^[^ ^73^ ^]^ 9464D cells were cultured in DMEM containing 10% FBS (R&D Systems), 1% nonessential amino acids, 0.5% antibiotic‐antimycotic, and 0.05% *β*‐mercaptoethanol.

### PBNP Synthesis

PBNPs were synthesized using a scheme as described previously.^[^ ^9^ ^]^ Briefly, citric acid was added to both aqueous solutions of FeCl_3_·6H_2_O (10 × 10^−3^ m; 20 mL) and K_4_[Fe(CN)_6_]·3H_2_O (10 × 10^−3^ m; 20 mL), separately, at 400 to 600 rpm at 60 °C to a final concentration of 5 × 10^−3^ m in each solution. Once dissolved, the K_4_[Fe(CN)_6_]·3H_2_O solution was added to the Fe(Cl)_3_·6H_2_O solution dropwise under stirring at 400 to 600 rpm. After stirring for 5 min at room temperature (no heat), the resulting particles were isolated by centrifugation in equal volumes of acetone and water (10 000 × *g* for 15 min at RT), and then rinsed/resuspended by microtip sonication (40% amplitude for 30 s) in Milli‐Q water using a Q500 sonicator (QSonica LLC, Newton, CT, USA) with a standard probe (#4220). Isolation and rinse steps were repeated three times before the particles were finally resuspended in Milli‐Q water with sonication at the desired concentration. PBNPs were analyzed for concentration by oven‐drying particles at 80 °C and measuring absorbance at 680 nm using SpectraMax microplate reader (Molecular Devices, San Jose, CA). The size distributions and surface charge of the PBNPs were characterized by dynamic light scattering on a Zetasizer Nano ZS (Malvern Instruments, Malvern, UK).

### Laser Delivery for PTT

For I‐PTT, an SMA‐905 fiber coupler (Laserglow Technologies, Toronto, Canada) was mounted onto a near‐infrared (NIR), collimated diode laser system (808 nm; Laserglow Technologies). The fiber coupler's front plate was adjusted to maximize the output power of the converged laser light. The optical fibers were then attached to the SMA‐905 end of the fiber coupler for interstitial laser delivery in the studies (Figure S1a, Supporting Information). The optical fibers were fitted with a cylindrical terminal diffuser 5 mm in length (LifePhotonic, Bonn, Germany) or with a flat‐cut end (Thorlabs, Newton, NJ) (Figure S3, Supporting Information). Both diffuser and flat‐cut optical fibers had a numerical aperture NA = 0.22 and core diameters of 200 µm, with the cladding increased actual diameter to 500 µm. This output power was measured and confirmed for studies using the IS6‐D‐Vis (for divergent beams) (part number 7Z02488, Ophir Optronics, LLC, North Logan, UT, USA) detector connected to the Juno RHS meter (part number 7Z01250, Ophir), which was then connected to a computer for output power reading using StarLab v3.62 software. For the I‐PTT studies in vitro, the fibers were inserted into the test suspensions (containing nanoparticles and/or tumor cells), with the middle of the diffuser or the end of the flat‐cut fiber submerged halfway in the cell solution, when using the diffuser and flat‐cut optical fibers, respectively (Figure S1b, Supporting Information). The fibers were attached to clamp stands to set and stabilize the fiber position within the test solution.

For S‐PTT with the NIR laser, the laser unit was attached to a clamp and onto a clamp stand, with the laser beam being applied externally (from the top) to the cell suspension (below). In these studies, the output laser power of the external beam was confirmed using a power meter (PM100D, Thorlabs, Newton, NJ, USA). The spot size of the beam was 5 mm by 8 mm (40 mm^2^).

### In Vitro PBNP‐PTT

For the photothermal heating studies, PBNPs were suspended in 500 µL of 1× PBS, 2% agarose gel (Sigma‐Aldrich) in deionized water, or 3.0 mg mL^–1^ Matrigel in 1X PBS (Corning, Washington, D.C., USA) to a final concentration of 0.1 mg mL^–1^ PBNPs. The samples were then illuminated using the NIR laser for 10 min at 0.4 and 0.6 W output laser power in both I‐PTT and S‐PTT configurations. To assess the effect of PBNP‐PTT on tumor cells, 5 million N2A cells were resuspended in 500 µL 1X PBS with or without 0.1 mg mL^–1^ PBNPs, or 5 million 9464D cells were resuspended in 500 µL 1X PBS with or without 0.15 mg mL^–1^ PBNPs. The samples were then illuminated using an NIR laser for 10 min at varied laser powers to generate similar thermal doses between I‐PTT and S‐PTT treatment groups.

Temperature distributions of the nanoparticle and cell suspensions were measured and recorded every minute for 10 min using a thermal camera (FLIR E5‐XT, Arlington, VA). The thermal doses administered were calculated and represented as the logarithm of the cumulative equivalent minutes at 43 °C (log(CEM43)) over 10 min, with the CEM43 calculated as previously described and shown in Equation 2.^[^ ^45^ ^]^

(2) $$\text{CEM43}=\sum_{i=1}^{n}t_{i}*R^{\left(43-T_{i}\right)}$$

Briefly, *t*_i_ is the *i*‐th time interval which is per minute, *n* is the number of time intervals which is 10 min, *T* is the average temperature during that time interval per minute, and *R* is a temperature‐dependent constant with a value of 0.25 for *T* < 43 °C and 0.5 for *T* > 43 °C. Since thermal doses were expressed as a logarithm of CEM43, a positive thermal dose indicated that a sample attained a temperature of at least 43 °C for a length of time greater than 1 min. By contrast, a negative thermal dose indicated that a sample failed to achieve 43 °C for at least 1 min while a thermal dose of exactly 0 indicated that a sample achieved a temperature of 43 °C for exactly 1 min. The PTT‐treated cells were then plated in 6‐well plates and incubated at 37 °C for 24 h, followed by downstream cellular analysis.

### Cellular Analyses for Viability and Immunogenic Cell Death In Vitro

To compare the effect of S‐PTT and I‐PTT on cells, cells were assessed for viability to determine the extent of cell death by each treatment. Additionally, samples were further analyzed to determine if ICD was elicited by measuring their biochemical correlates, including surface expression of calreticulin and release of ATP and HMGB1.^[^ ^48^ ^]^

### Flow Cytometry

After treatment with PTT or controls as described above and followed by incubation at 37 °C, the tumor cells were harvested and stained with Zombie Violet Fixable viability dye (Biolegend, 423114). Cells were then blocked using a TruStain FcX anti‐mouse CD16/32 (clone 93, 101320; Biolegend) antibody to prevent nonspecific binding of Fc receptors during surface staining. After Fc blocking, cells were then surfaced stained with a fluorescent antibody against calreticulin (Abcam, ab209577).^[^ ^48^ ^]^ Flow cytometry was performed using the BD Biosciences Celesta Cell Analyzer (Franklin Lakes, NJ), and analyzed with the FlowJo software (Ashland, OR). Both stains were done at 1:100 dilution in PBS or staining buffer.

### ATP Analysis

To determine the decrease in intracellular ATP, the treated cells were harvested, washed at 24 h after PTT treatments, and then mixed with ATP reagent from the CellTiter‐Glo assay (Promega, Madison, WI), according to the manufacturer's specifications. Briefly, equal volumes of cell suspension and ATP reagent were added to an opaque 96‐well plate and then mixed using a plate shaker for 2 min for effecting cell lysis and followed by a 10 min incubation in the dark for stabilizing the luminescent signal. Luminescence was measured using a SpectraMax i3x microplate reader (Molecular Devices, LLC. San Jose, CA). Results were expressed as a percentage of intracellular ATP measured in vehicle‐treated, control cells.

### HMGB1 Release Analysis

After incubation for 24 h, cell supernatants from the various treatment groups were collected by centrifugation at 400 ×*g* for 5 min. These supernatants were centrifuged a second time at 10 000 ×rpm for 3 min at 4 °C to remove insoluble materials and lipids. The resulting supernatants were collected and stored at −80 °C until analysis. For analysis, supernatants were measured for released HMGB1 using the HMGB1 Detection ELISA kit (Chondrex, Inc.; Woodinville, WA), according to the manufacturer's instructions. Briefly, 96‐well ELISA plates were coated with HMGB1 antibody overnight at 4 °C. The supernatant samples and those from an HMGB1 standard were diluted 1:1 with sample dilution buffer and added to the coated ELISA plates in duplicate. The detection antibody solution was added and then incubated at 37 °C for 1 h. Subsequently, the plates were incubated overnight at 4 °C. Next, the plates were washed and wells were then incubated with streptavidin peroxidase solution for 30 min at room temperature. The wells were then washed and incubated with 3,3′,5,5′‐tetramethylbenzidine solution for 30 min at room temperature. After incubation, the stop solution was added and absorbance was measured at 450 nm using the SpectraMax i3X microplate reader. Media alone was used as a blank for all conditions. The appropriate blanks were subtracted from the standards and samples to generate an HMGB1 standard curve to accurately estimate the concentration of HMGB1 released from the different treatment groups in ng mL^–1^.

For cell viability analysis of 9464D cells in 2% agar gel phantoms, 2 to 5 million 9464D cells were suspended with 2% agar gel heated to ≈40 °C containing 0.15 mg mL^–1^ PBNP in serum‐free DMEM media, in Eppendorf tubes. Once the agar gels solidified by cooling, I‐PTT or S‐PTT was conducted as described above. Temperatures were monitored using the thermal camera as described above. After treatment, gel‐embedded cells were incubated at 37 °C for 3–4 h. The gels were then removed from the tubes, and the inner 40 mm section of the gel (Figure 4a) was cut for sectioning. Sectioning was completed using the Leica VT1000 S Fully Automatic Vibrating Blade Microtome from the GW Nanofabrication and Imaging Center. 100 µm sections were obtained from the initial 40 mm section of gel‐embedded cells and stained 1:500 in PBS with calcein AM (Invitrogen, Waltham, MA) and DAPI for 5–6 min at room temperature protected from light by covering with foil. Sections were then washed 1x with PBS and then placed on a microscope slide. Fluorescent microscope images were obtained using the Revolve ECHO microscope (ECHO, San Diego, CA, USA) and the ECHO Pro, Echo laboratories application software provided by Apple Inc. (Cupertino, CA, USA). The numbers of live or dead cells on the acquired fluorescence microscopy images were determined using the particle analysis plugin on ImageJ (https://imagej.nih.gov/ij/).

### FEM Simulations for Intratumoral Light Propagation

The FEM approach described in Shafirstein et al. 2004, Oakley et al. 2015, and Shafirstein et al. 2018 was applied to model intratumoral light propagation.^[^ ^49^, ^50^, ^51^ ^]^ Briefly, FEM was employed to solve the 3D time‐dependent light diffusion approximation as derived from the equation for radiative transport. The light irradiance (mW cm^–2^) was computed throughout a 5 × 5 × 3 mm tumor model that represented a tumor containing PBNP nanoparticles and illuminated with 0.75 W of 808 nm laser light at the skin surface with a spot size of 5 × 8 mm (S‐PTT, Figure S8a, Supporting Information) or with 0.45 W of 808 nm laser light delivered through an optical fiber with a 5 mm cylindrical diffuser end. The effects of PBNP nanoparticles on the optical properties were included by assuming that the absorption and reduce scattering coefficients were proportional to the concentration of PBNP, consistent with the approach used in Shafirstein et al. for adding an exogenous light absorber to tissue.^[^ ^74^ ^]^ The volume fraction of PBNP in the tumor model was set to 0.4 and the nanoparticles were assumed to be uniformly distributed within the tumor model. For simulating laser light propagation in S‐PTT, it was assumed that 20% of the light is reflected from the surface. For simulating light propagation in I‐PTT, the light was modeled as a flux of photons emitted from the inside of a catheter (inner diameter of 0.98 mm; outer diameter of 1.3 mm) inserted through the center of the tumor model (Figure S8b, Supporting Information). The optical properties used in these simulations are shown in Table S1 in the Supporting Information.

### Animals

All animal studies were conducted in accordance with protocols approved by the Institutional Animal Care and Use Committee of the George Washington University (protocol # A2021‐001) and in accordance with the humane care of research animals. For tumor growth and survival studies, 5 weeks old female A/J mice and C57BL/6 mice were purchased from Jackson Laboratory (Bar Harbor, ME) and acclimated for a week. For tumor rechallenge studies, age‐matched or 10 weeks old female A/J mice and C57BL/6 mice were acquired for Jackson Laboratory for each respective tumor model.

### Mouse Neuroblastoma Models

For the single tumor models, syngeneic neuroblastoma mouse models were established by suspending 1 million cells of N2a or 9464D cells in 100 µL PBS and subcutaneously injected into the shaved backs of 6–7 weeks old female A/J (N2A model) or C57BL/6 mice (9464D model), respectively. Tumors were treated once volumes reached a size of 5 mm (44–60 mm^3^).

For the metachronous two‐tumor model, 1 million 9464D cells in 100 µL PBS were subcutaneously injected 7 days apart on contralateral sides of the shaved backs of 6–7 weeks old female C57BL/6 mice. The tumor that reached a size of 5 mm (70 mm^3^) first (i.e., the larger tumor) was selected as the primary tumor and treated with PBNP‐PTT. The contralateral tumors (designated as the secondary tumor) were left untreated to evaluate the systemic effect of the treatments. Animals were monitored periodically and tumor volume (mm^3^) was calculated by measuring tumor dimensions (length and width) and using the formula: (length x width^2^)/2.

### In Vivo PBNP‐PTT

Mice were anesthetized prior to treatment using 2–5% isoflurane. For PBNP‐PTT, tumors designated for treatment were intratumorally (i.t.) injected with PBNPs (50 µL of 1 mg mL^–1^ PBNPs). For S‐PTT treatments, the tumors were then exposed to the external NIR laser beam for 10 min at ≈0.75 W, and the power was adjusted over the treatment period to maintain a superficial temperature range, as specified for each in vivo study. For I‐PTT Diffuser, a shielded IV catheter (inner diameter of 0.98 mm and outer diameter of 1.3 mm, BD biosciences) was first placed into the core of the tumor. The catheter was then secured and stabilized using medical tape. The optical fiber with a 5 mm terminal cylindrical diffuser (which was coupled to the NIR laser as described above) was then inserted through the catheter so that the terminal diffuser was positioned within the tumor (Figure S1c, Supporting Information). Once the fiber was properly placed, the laser was turned on and the laser power was adjusted accordingly to attain desired treatment temperatures at the treatment zone covering the tumor, with tumors treated for 10 min. Temperature was monitored with the thermal camera, and laser power was adjusted over the treatment period to maintain a superficial temperature range, as specified for each in vivo study.

### In Vivo Studies

In the single tumor study, when the injected tumors reached a dimension of 5 mm in length or width (44–60 mm^3^ in volume), the animals were randomly distributed into the various treatment groups. For single tumor studies, the mice were divided into the four groups: 1) Untreated (receiving no treatment), 2) S‐PTT (receiving i.t. injection of 50 µL of 1 mg mL^–1^ PBNPs and irradiated with external NIR laser to measured temperatures of 60 and 70 °C for the N2A and 9464D tumors, respectively), 3) I‐PTT Diffuser (receiving i.t. injection of 50 µL of 1 mg mL^–1^ PBNPs and irradiated with the optical fiber with a 5 mm terminal diffuser coupled to the NIR laser to temperatures of 60 °C and at least 60 °C for the N2A and 9464D tumors, respectively). The mice were treated for 10 min, with temperatures measured and recorded using the thermal camera every minute. After treatment, the mice, tumor sizes, and tumor‐free and overall survival were monitored and recorded daily. Tumor‐free treated mice were rechallenged at Day 80 post‐treatment with subcutaneous injection of 1 million N2A or 9464D cells in 100 µL PBS in the opposite flank, and monitored for tumor growth, respective for each tumor model. Age‐matched female A/J and C57BL/6 mice that had not previously been injected with N2A or 9464D cells served as “treatment naïve” controls for rechallenge studies.

In the metachronous two‐tumor study, tumor‐bearing mice were divided into the following three groups, with the 9464D primary tumor treated accordingly: 1) Vehicle (administered PBS into the tumor), 2) S‐PTT (receiving i.t. injection of 50 µL of 1 mg mL^–1^ PBNPs and irradiated with external NIR laser to measure temperatures of 100 °C), and 3) I‐PTT Diffuser (receiving i.t. injection of 50 µL of 1 mg mL^–1^ PBNPs and irradiated with the optical fiber with a 5 mm terminal diffuser coupled to the NIR laser to final tumor temperatures of at least 60 °C). The mice were treated for 10 min, with temperatures measured and recorded using the thermal camera every minute. Mice were monitored for tumor relapse at the primary tumor treated site and for tumor progression/regression on the secondary, untreated tumor side.

Mice were humanely euthanized according to the approved IACUC protocol, through CO_2_ narcosis followed by cervical dislocation, when tumor diameters measured 20 mm or no later than a week after tumor ulcerations were first observed.

### Hemolysis

Blood was collected from healthy mice via cardiac puncture into tubes coated with 1 mg of K2EDTA. Blood was centrifuged at 1500 rpm for 15 min at 4 °C to remove plasma and pellet the red blood cells. The cells were then resuspended in PBS. 100 µL of blood was aliquoted into separate tubes. PBNPs diluted in PBS were added at final concentrations of 0.0625, 0.125, 0.25, or 0.5 mg mL^–1^ in a total volume of 200 µL. For the positive control, Triton X‐100 was added at 0.5%. Samples were incubated at 37 °C for 1 h. Red blood cells were then pelleted by centrifugation at 400 x*g* for 5 min. Digital images were captured after centrifugation. Absorbance at wavelengths from 500 to 700 nm was measured using the SpectraMax i3X microplate reader.

### ALT and AST Activity Assay for Liver Function Analysis

50 weeks old healthy mice were injected with PBNP at 2.5 mg kg^–1^. Blood was collected at Day 3 and Day 24 by submandibular blood drawing into ethylenediaminetetraacetic acid‐free Eppendorf tubes. Blood was allowed to clot at room temperature for 30 min. Sera from these samples were then collected by centrifugation at 1000 x*g* for 15 min at 4 °C. The collected sera were stored at −80 °C until analysis. The ALT activity was evaluated using the colorimetric ALT activity assay kit (abcam, Cambridge, UK). The protocol from the manufacturer was followed using a 1:10 dilution of the sera. Absorbance at 570 nm was measured by SpectraMax i3X microplate reader. The AST activity was evaluated using the AST activity assay kit (Sigma‐Aldrich). The protocol from the manufacturer was followed using a 1:10 dilution of the sera. Absorbance at 450 nm was measured using the SpectraMax i3X microplate reader.

### Statistical Analysis

1) Preprocessing of data: normalization was used in this study to represent the expression of intracellular ATP levels in the various treatment groups (as a percentage of the vehicle‐treated group). All animal studies were performed in a nonblinded fashion, and mice that attained similar average volumes of the primary tumors were randomized into the various treatment groups. All other data acquired in this study were presented as acquired without any transformation or exclusion of outliers. 2) Data presentation: results obtained in this study were expressed as mean ± standard deviation. 3) Sample size (*n*) for each statistical analysis: all in vitro studies had a sample size of *n* = 3 unless specifically noted for a study. For the in vivo studies, with the exception of the rechallenge studies where only the surviving long‐term surviving mice and the two tumor mouse study in the 9464D model, all in vivo studies had a sample size of at least 6. 4) Statistical methods: Statistical significance between treatment groups was determined using one‐way and two‐way analysis of variance (ANOVA), and values with *p* < 0.05 were noted as statistically significant. Survival results were analyzed according to Kaplan–Meier curves, and log‐rank tests were used to determine the statistically significant differences among survival groups. Data were approximated as normally distributed assuming variance was similar between the treatment groups. Statistical significance throughout the study was indicated as *p < 0.05, **p < 0.01, ***p < 0.001, and ****p < 0.0001. 5) Software used for statistical analysis: analysis was performed using the Prism 8.1.1 software (GraphPad).

## Conflict of Interest

The authors declare no conflict of interest.

## Supporting information

Supporting Information

## Data Availability

The data that support the findings of this study are available from the corresponding author upon reasonable request.
