# Supplementary material for: Interstitial Photothermal Therapy Generates Durable Treatment Responses in Neuroblastoma
Source: Adv Healthc Mater. 2022 Aug 18;11(20):2201084. doi: 10.1002/adhm.202201084 (PMC9588730; doi:10.1002/adhm.202201084)
Supplement: Supplementary file 1 — Supporting Information [file ADHM-11-2201084-s001.pdf]

ADVANCED HEALTHCARE MATERIALS

Supporting Information

for *Adv. Healthcare Mater.*, DOI 10.1002/adhm.202201084

Interstitial Photothermal Therapy Generates Durable Treatment Responses in Neuroblastoma

*Debbie K. Ledezma, Preethi B. Balakrishnan, Anshi Shukla, Jacob A. Medina, Jie Chen, Emily Oakley, Catherine M. Bollard, Gal Shafirstein, Mario Miscuglio and Rohan Fernandes**

WILEY-VCH

Supporting Information

Interstitial Photothermal Therapy Generates Durable Treatment Responses in Neuroblastoma

*Debbie K. Ledezma, Preethi B. Balakrishnan, Anshi Shukla, Jacob A. Medina, Jie Chen, Emily Oakley, Catherine M. Bollard, Gal Shafirstein, Mario Miscuglio, Rohan Fernandes**

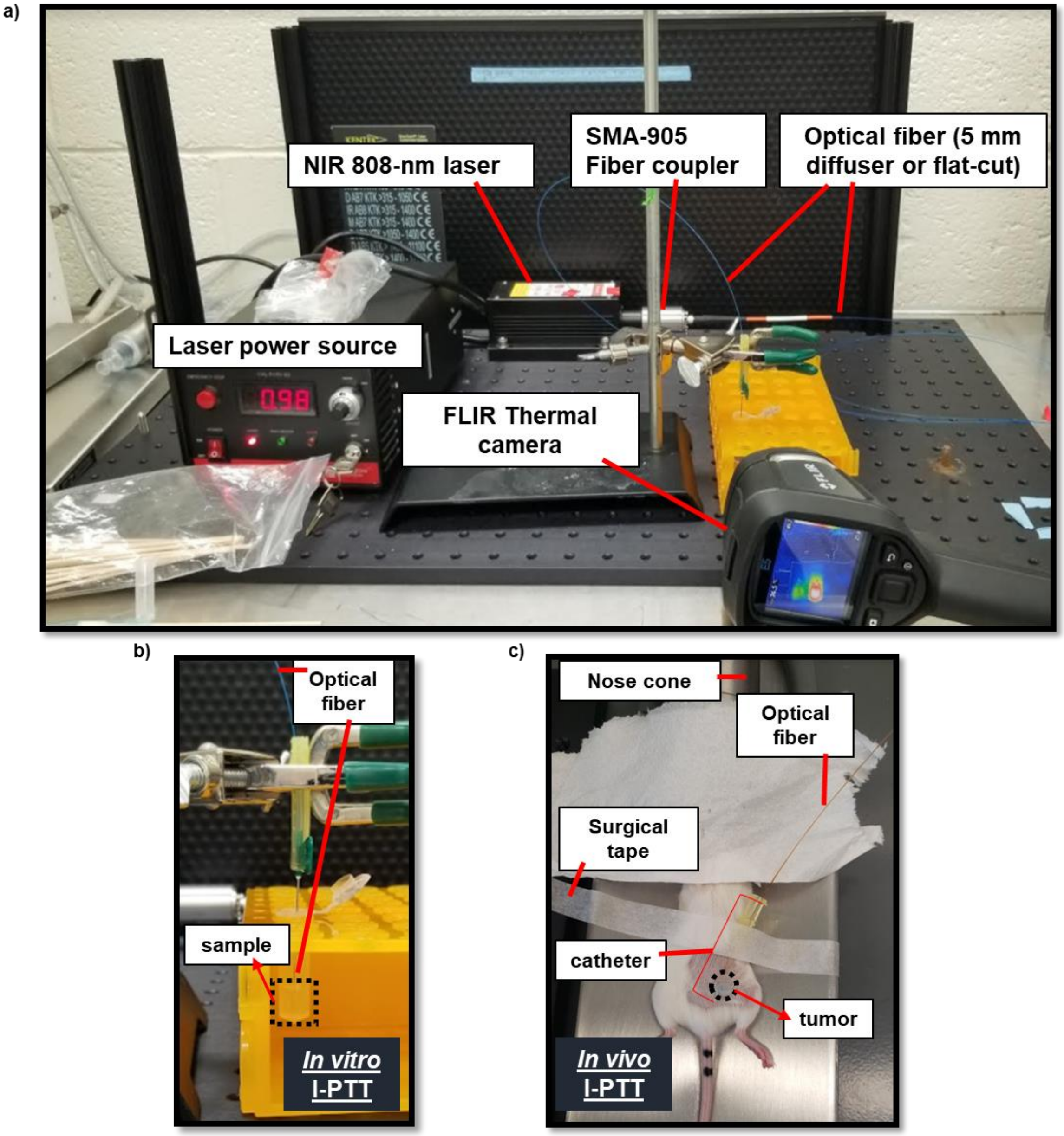

Figure S1. Photograph of the laser system utilized to administer interstitial PTT. a) An 808 nm near-infrared (NIR) continuous wave, collimate diode laser with its power source was fixed onto an optical breadboard. An SMA-905 fiber coupler was attached to the end of the NIR laser, and optical fibers (with a terminal 5 mm diffuser or flat-cut end) with appropriate SMA-905 connectors were attached to the other side of the coupler. The fibers were stabilized on a stand with surgical tape and with a clamp holding a catheter or pipet tip to stabilize, orient, and place the fiber into a test sample. Samples were typically placed within Eppendorf tubes for these studies, with the tubes placed on a 4-way tube rack. A thermal camera was positioned facing the tube through the tube rack opening to measure and record temperature and thermal images every minute or as needed for each study. b) I-PTT set-up for *in vitro* studies. c) I-PTT set-up for *in vivo* studies, with the catheter placed through the tumor and the optical fiber inserted into the tumor through the catheter.

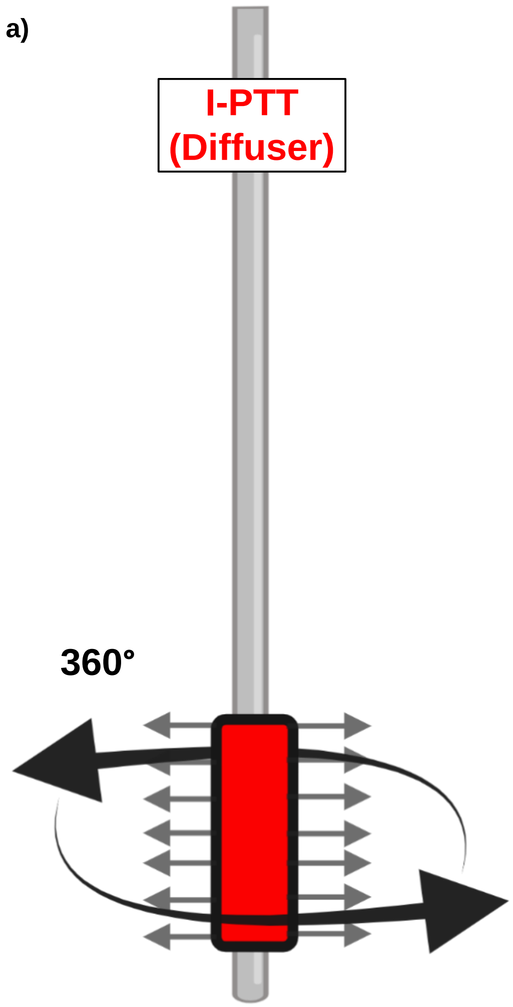

Figure S2. Pathway of laser light emitted from optical fibers use for I-PTT. a) The optical fiber with a terminal cylindrical diffuser end emits red laser light along its length (irradiation length: 5 mm) and around its circumference. b) The optical fiber with a flat-end cut emits red laser light from the end of the fiber in one direction with light illuminating as a diffused ball.

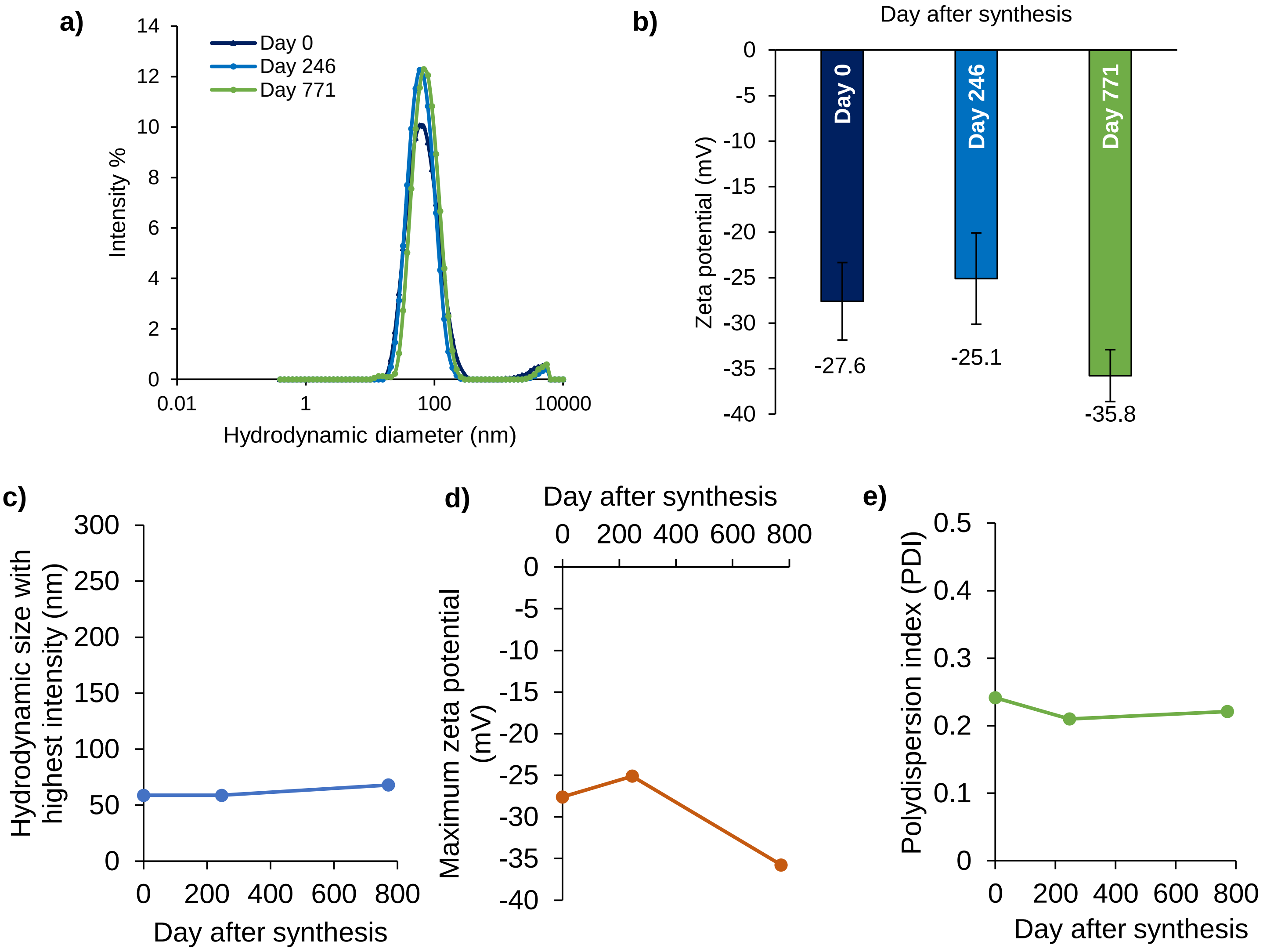

Figure S3. Prussian blue nanoparticles (PBNPs) used in the study exhibited long-term stability. a) Size distributions of PBNPs, based on the hydrodynamic diameters, ranged from 50-70 nm as measured by dynamic light scattering (DLS) on Days 0, 246, and 771 after synthesis. b) PBNPs maintained a negative zeta potential (surface charge) (< -25 mV) over several days, as measured by DLS. c) Size of PBNPs over time. d) Zeta potential over time. e) Polydispersity index (PDI) over time.

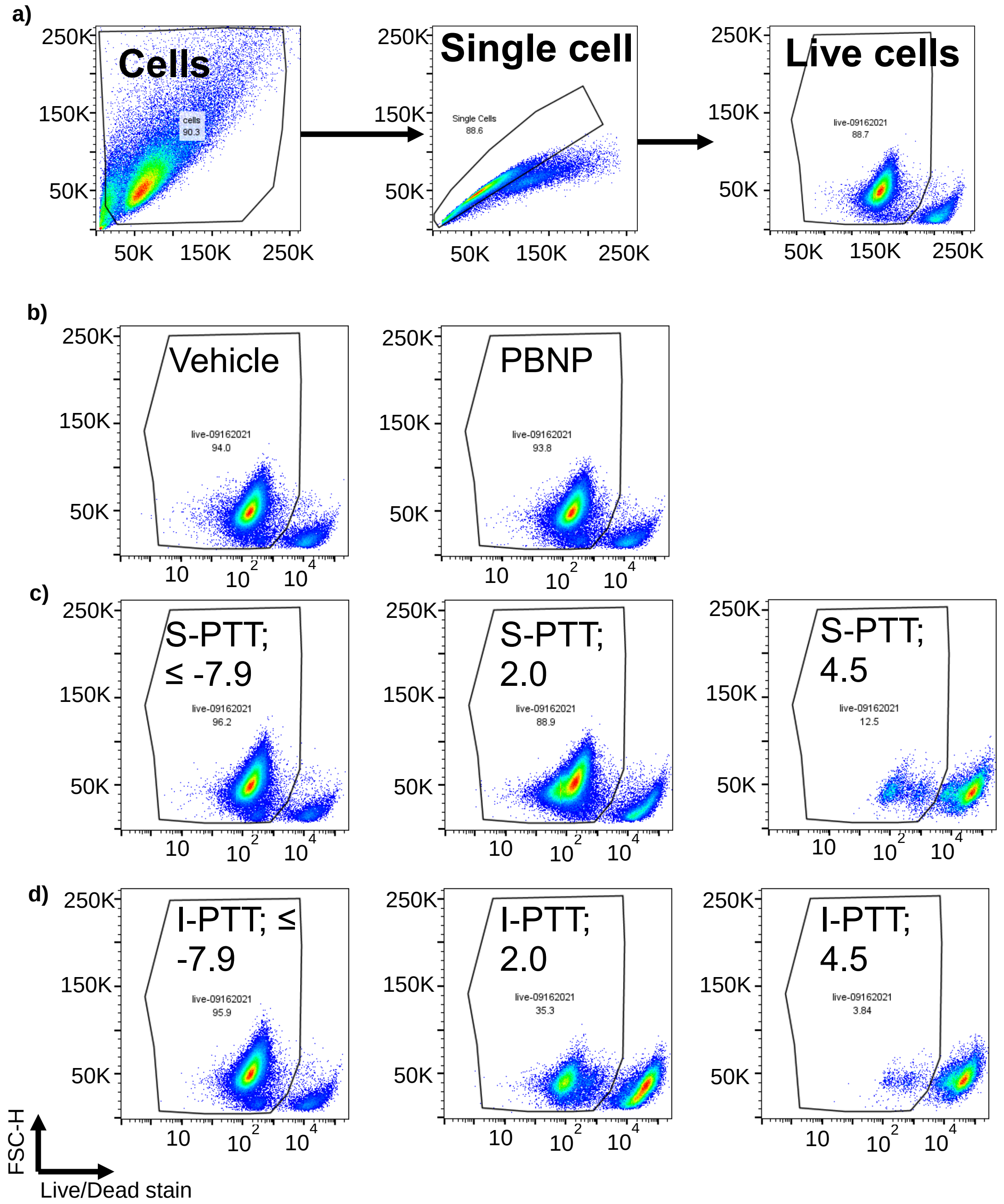

Figure S4. Flow cytometry analysis of N2A cells measuring cell viability post-PTT and control treatments. a) The gating strategy used to assess live cells post-PTT. b-d) Flow scatter plots of live N2A cells after b) control treatments, c) S-PTT and d) I-PTT treatments at the noted thermal doses, demonstrated a decreasing proportion of live cells with increasing thermal dose.

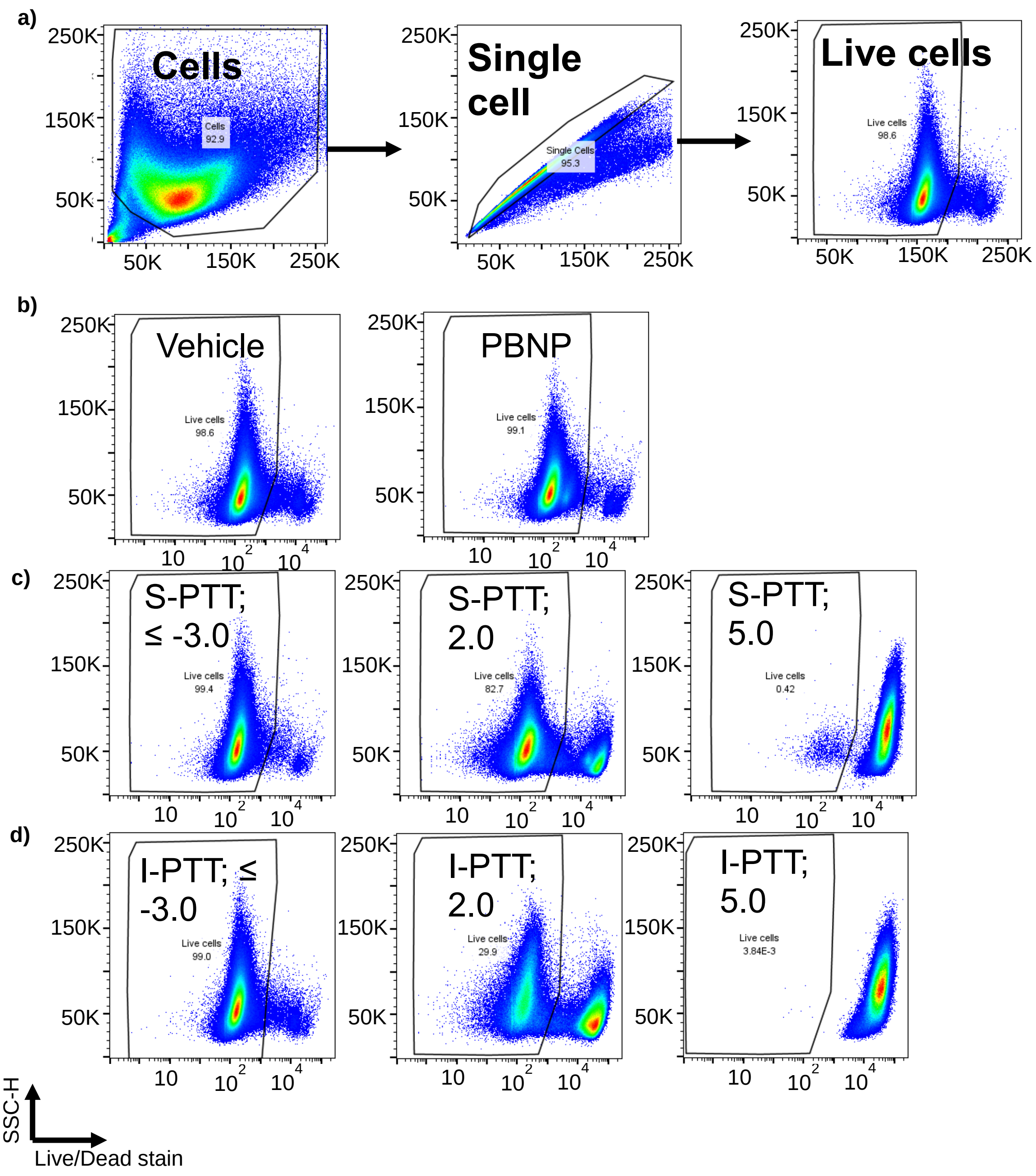

Figure S5. Flow cytometry analysis of 9464D cells measuring cell viability post-PTT and control treatments. a) The gating strategy used to assess live cells post-PTT. b-d) Flow scatter plots of live 9464D cells after b) control treatments, c) S-PTT and d) I-PTT treatments at the noted thermal doses, demonstrated a decreasing proportion of live cells with increasing thermal dose.

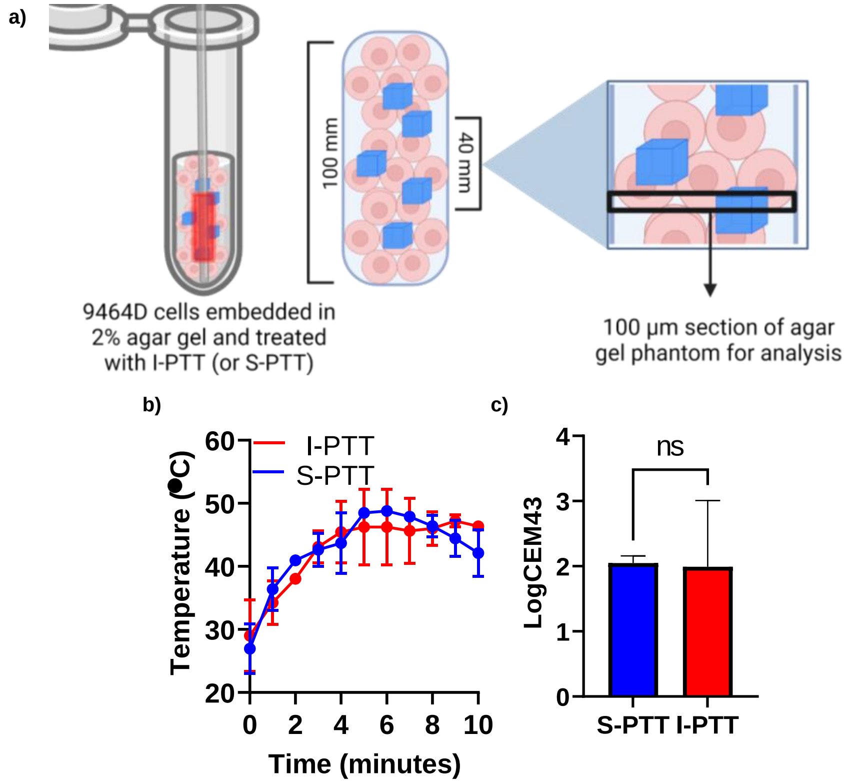

Figure S6. Treatment of 9464D in agar gel phantoms. a) Schematic of I-PTT (or S-PTT) administered and analysis of 9464D viability in 2% agar gel phantoms. b) Temperature and thermal doses for 9464D cells treated in 2% agar gel phantoms. N=2.

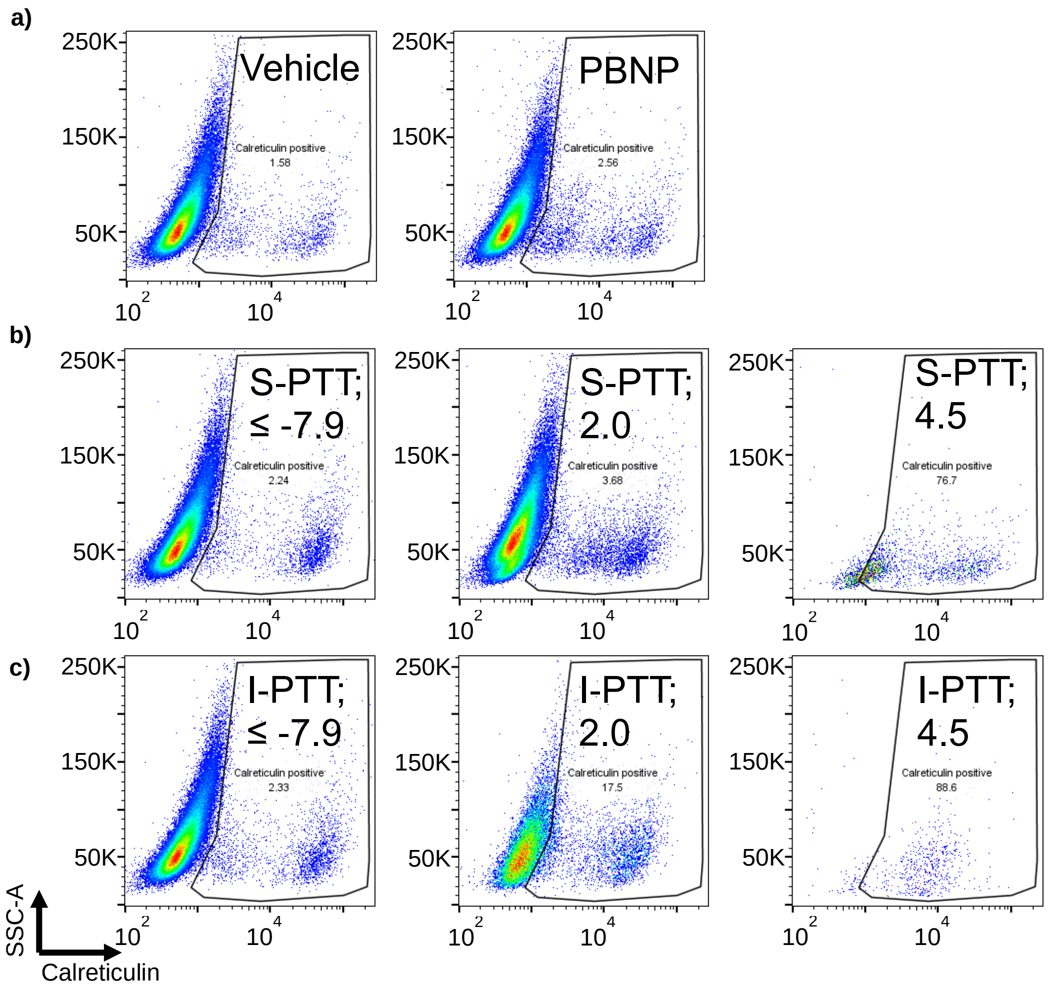

Figure S7. Surface calreticulin expression on N2A cells analyzed by flow cytometry. Flow scatter plots of N2A cells showing the percentage of surface calreticulin-expressing cells, gated on live cells, after a) control treatments (vehicle-treated and PBNP-treated), b) S-PTT treatments, and c) I-PTT treatments at the noted thermal doses. Calreticulin expression increased with higher thermal doses, for both I-PTT and S-PTT.

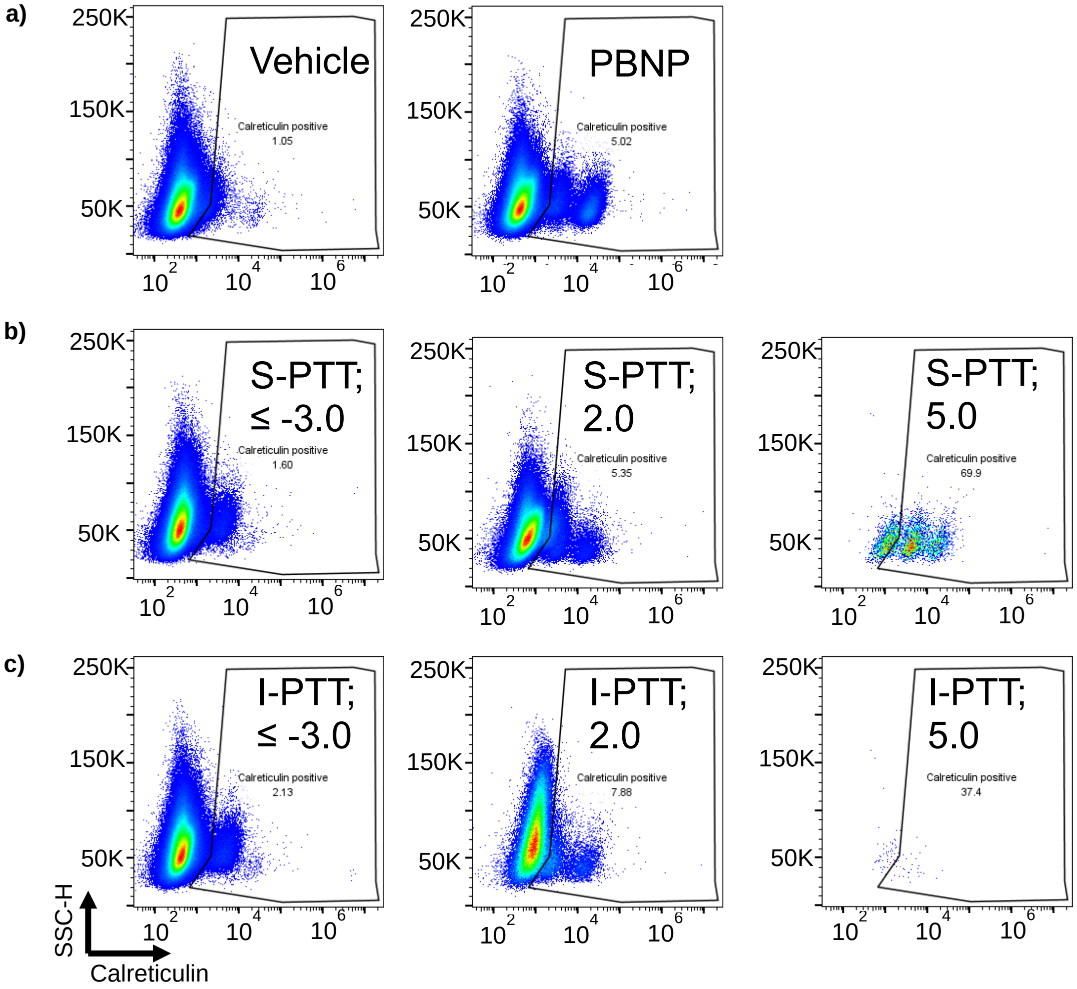

Figure S8. Surface calreticulin expression on 9464D cells analyzed by flow cytometry. Flow scatter plots of 9464D cells showing the percentage of surface calreticulin-expressing cells, gated on live cells, after a) control treatments (vehicle-treated and PBNP-treated), b) S-PTT treatments, and c) I-PTT treatments at the noted thermal doses. Calreticulin expression increased with higher thermal doses, for both I-PTT and S-PTT.

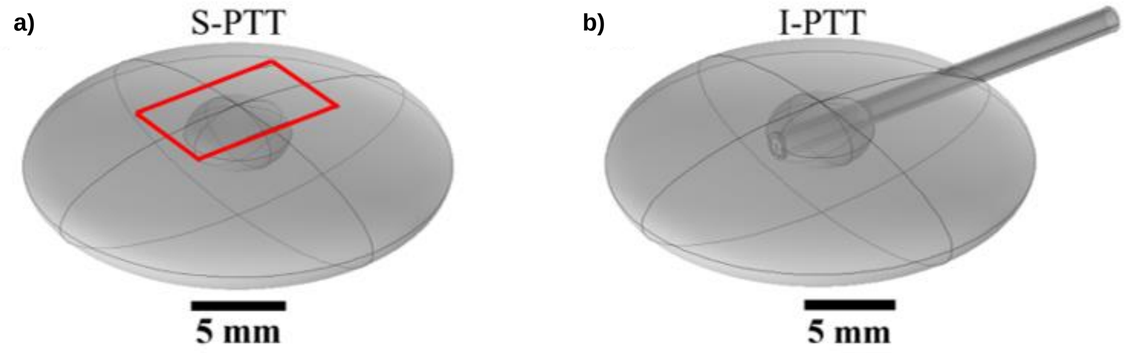

Figure S9. FEM Simulations of light propagation during S-PTT and I-PTT. a) Geometry for S-PTT. b) Geometry for I-PTT. For both geometries, the tumor was modeled as an ellipsoid with a length and width of 5 mm and a depth of 3 mm. A larger ellipsoid with a length and width of 10 mm and a depth of 6 mm was created to represent the surrounding normal tissue. For S-PTT, the light distribution was computed for 808-nm laser light at a power of 0.75 W and a rectangular spot size of 5 mm x 8 mm (shown in red in fig. a). For I-PTT, an optical fiber with cylindrical diffuser end of 5 mm was used to illuminate the tumor with 808-nm laser light at a power of 0.45 W. The cylindrical diffuser was inserted into the center of the tumor as shown in Fig. b.

Supporting Table 1. Optical Properties for S-PTT and I-PTT Simulations

	Input Data	Description
Tumor Tissue and Surrounding Normal Tissue	μ_a = 1612 (1/cm)<br>μ'_s = 7.6 (1/cm)<br>n = 1.37	Tissue linear absorption coefficient<br>Tissue reduced scattering coefficient<br>Tissue refractive index
Catheters for I-PTT	μ_a = 0.0001 (1/cm)<br>μ'_s = 0.002 (1/cm)<br>n = 1.45	Catheter linear absorption coefficient<br>Catheter reduced scattering coefficient<br>Catheter refractive index

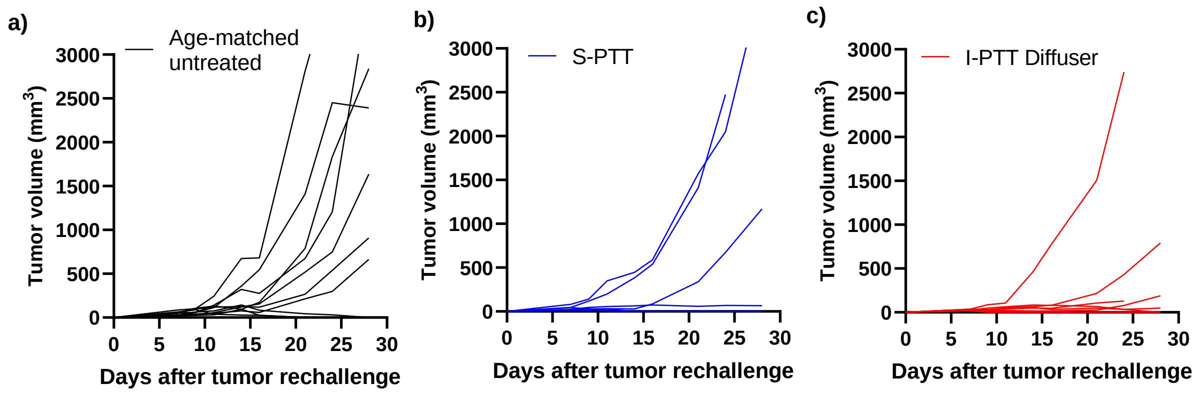

Figure S10. Tumor growth curves of N2A rechallenge tumors. Individuals tumor growth curves of N2A rechallenge tumors of N2A tumors in mice that were previously a) untreated, b) treated with S-PTT, or c) treated with I-PTT. n=5-9.

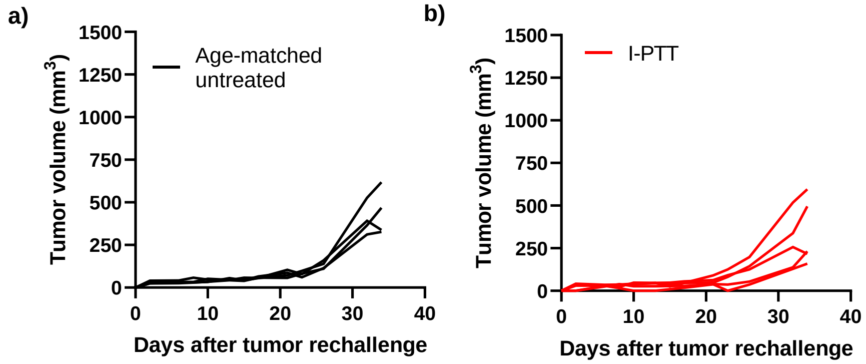

Figure S11. Tumor growth curves of 9464D rechallenge tumors. Individuals tumor growth curves of 9464D rechallenge tumors in mice that were previously a) untreated or b) treated with I-PTT. n=4-5.

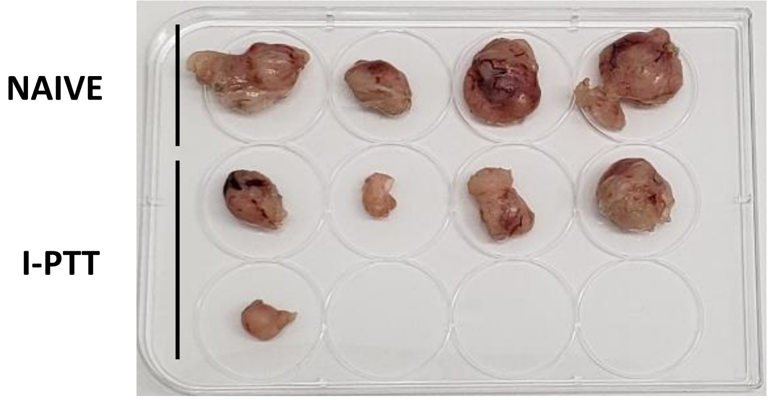

Figure S12. Rechallenge tumor volumes of PTT-treated 9464D mice measured ex vivo. Photographs of tumors from naïve tumor-bearing mice and I-PTT-treated mice at Day 40 after the rechallenge inoculation.

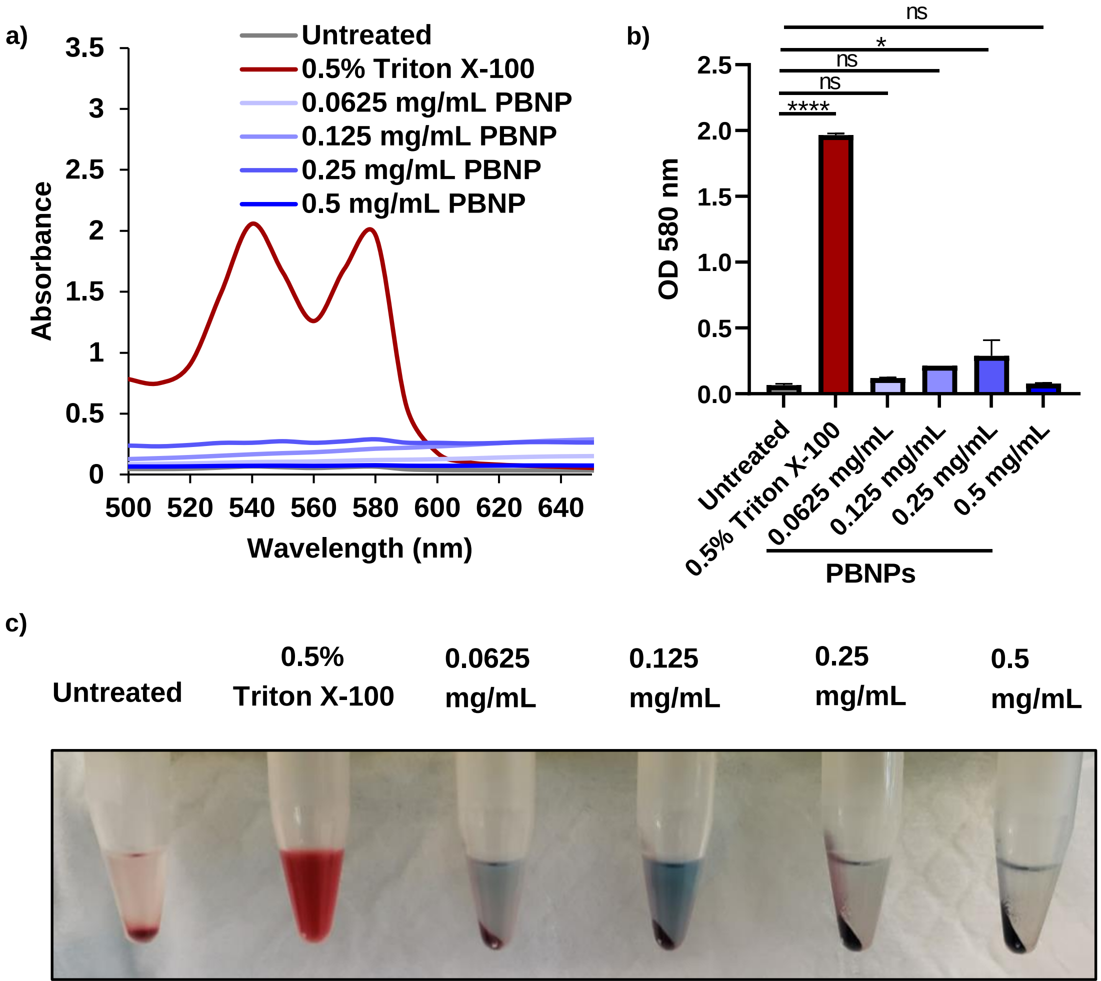

Figure S13. Hemolysis of PBNP-injec

ted healthy mice compared to untreated healthy mice. a) Absorbance spectrum of cell-free hemoglobin from 500 to 690 nm as a measure of hemolysis after red blood cells were incubated with varying concentrations of PBNPs at 37 ˚C for 1 hour. 0.5% Triton X-100 served as the positive control. b) Absorbance of cell-free hemoglobin at 580 nm. c) Representative image of supernatant of red blood cells after the noted treatments. Values represent means ± st.dev. n=2/group; ns: not significant, * $p < 0.05$, ** $p < 0.01$, *** $p < 0.001$, **** $p < 0.0001$ (ANOVA).

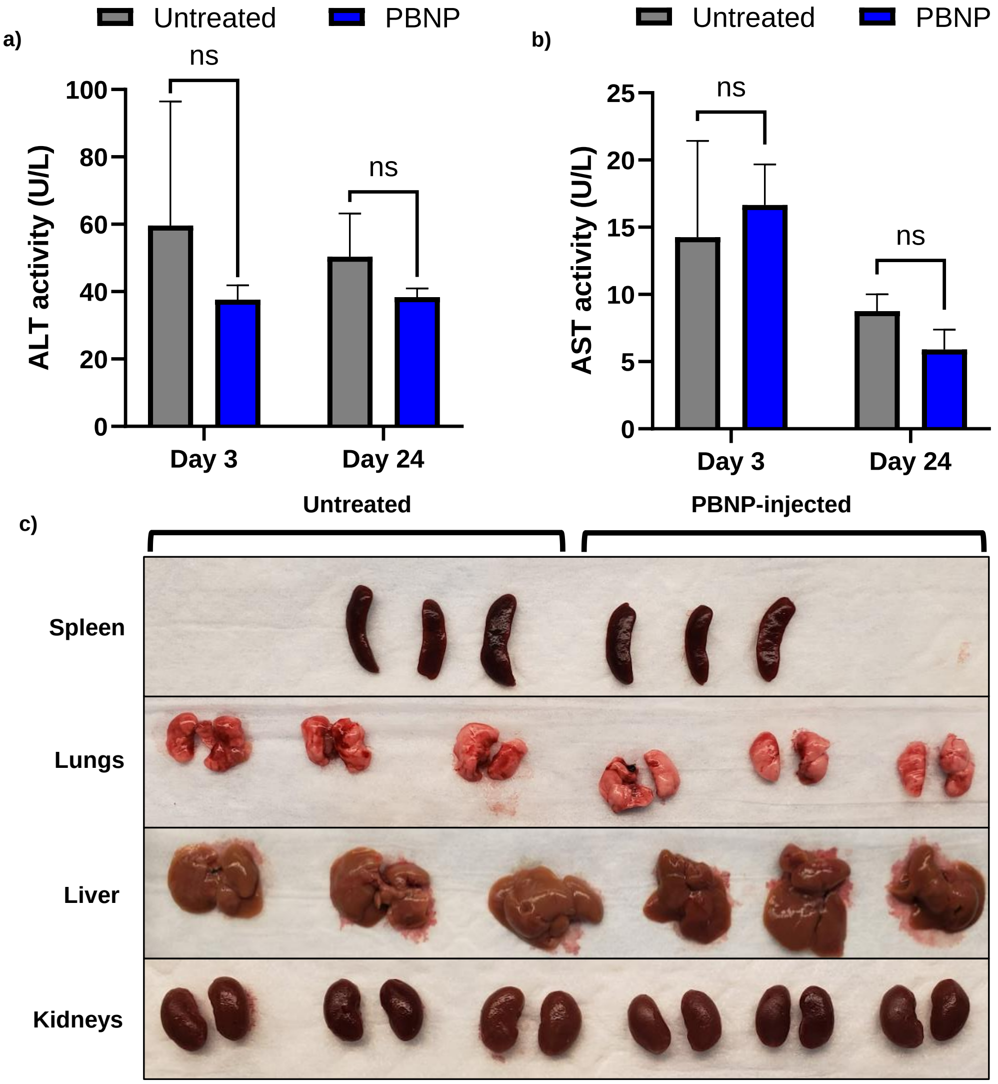

Figure S14. Liver enzyme evaluation and tissues of PBNP-injected 70-week old healthy mice compared to untreated healthy mice. a) ALT activity in PBNP-injected healthy mice compared to untreated healthy mice at Day 3 and Day 24 after PBNP injection. b) AST activity in PBNP-injected healthy mice compared to untreated healthy mice at Day 3 and Day 24 after PBNP injection. c) Representative images of spleen, lungs, liver, and kidneys of PBNP-injected healthy mice compared to untreated healthy mice at Day 36 after PBNP injection at the flank. Values represent means ± st.dev. n=3/group; ns: not significant, * $p < 0.05$, ** $p < 0.01$, *** $p < 0.001$, **** $p < 0.0001$ (ANOVA).
